# Supplementary material for: The Abdominal Pain Unit (APU). Study protocol of a standardized and structured care pathway for patients with atraumatic abdominal pain in the emergency department: A stepped wedged cluster randomized controlled trial
Source: PLoS One. 2022 Aug 24;17(8):e0273115. doi: 10.1371/journal.pone.0273115 (PMC9401147; doi:10.1371/journal.pone.0273115)
Supplement: S1 Checklist — (DOCX) [file pone.0273115.s001.docx]

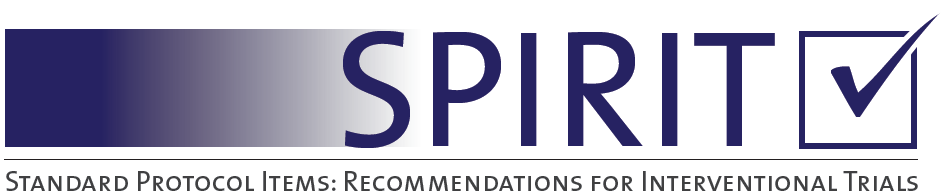


SPIRIT 2013 Checklist: Recommended items to address in a clinical trial protocol and related documents*

| Section/item | ItemNo | Description |  |
| --- | --- | --- | --- |
| **Administrative information** | |  |  |
| Title | 1 | Descriptive title identifying the study design, population, interventions, and, if applicable, trial acronym | The Abdominal Pain Unit (APU). Study protocol of a standardized and structured care pathway for patients with atraumatic abdominal pain in the emergency department: A stepped wedged cluster randomized controlled trial. |
| Trial registration | 2a | Trial identifier and registry name. If not yet registered, name of intended registry | German Clinical Trial Register (DRKS): DRKS00021052  <https://www.drks.de/drks_web/> |
|  | 2b | All items from the World Health Organization Trial Registration Data Set |  |
| Protocol version | 3 | Date and version identifier | May 2021, v1.1 |
| Funding | 4 | Sources and types of financial, material, and other support | German Federal Joint Committee’s (G-BA) Innovation Funds [grant number 01NVF19025] |
| Roles and responsibilities | 5a | Names, affiliations, and roles of protocol contributors | Prof. Dr. Martin Möckel,  Head of Emergency and Acute Medicine Campus Charité Mitte and Campus Virchow-Klinikum,  Charité - Universitätsmedizin Berlin, Campus Charité Mitte and Campus Virchow-Klinikum (CCM/CVK)  Phone: +49 (30) 450 - 553203;  Fax: +49 (30) 450 - 553203;  E-Mail: [martin.moeckel@charite.de](mailto:martin.moeckel@charite.de)  MM has received the funding from the German Federal Joint Committee’s (G-BA) Innovation Funds [grant number 01NVF19025] for the research proposal, had the idea and initiated the APU pathway. |
|  | 5b | Name and contact information for the trial sponsor | German Federal Joint Committee’s (G-BA) Innovation Funds |
|  | 5c | Role of study sponsor and funders, if any, in study design; collection, management, analysis, and interpretation of data; writing of the report; and the decision to submit the report for publication, including whether they will have ultimate authority over any of these activities | None. |
|  | 5d | Composition, roles, and responsibilities of the coordinating centre, steering committee, endpoint adjudication committee, data management team, and other individuals or groups overseeing the trial, if applicable (see Item 21a for data monitoring committee) | The project will be implemented under the consortium leadership of the Emergency and Acute Medicine, Campus Mitte und Virchow Klinikum Charité. The scientific evaluation is led by the Institute of Medical Sociology and Rehabilitation Science and the Institute of Health Services Research in Emergency Medicine together with the Institute of Medical Biometrics and Clinical Epidemiology and the Institute of Social Medicine, Epidemiology and Health Economics of the Charité–Universitätsmedizin Berlin. TMF e.V. is responsible for data protection aspects, such as the creation and coordination of the data protection concept, as well as for aspects regarding medical device regulations. |
| **Introduction** | | | |
| Background and rationale | 6a | Description of research question and justification for undertaking the trial, including summary of relevant studies (published and unpublished) examining benefits and harms for each intervention | A common complaint in patients presenting in the emergency department (ED) is atraumatic abdominal pain with a prevalence of 5 – 20 % per year [1, 2]. Patients with atraumatic abdominal pain have a very broad range of diagnoses and the associated hospital mortality rate of 5.1% is relatively high, e.g. compared to chest pain (with a 0.9% hospital mortality) [1]. Among patients aged 65 years or older, this mortality rate is even higher [3].  To assure high quality of care and potentially reduce mortality, it seems imperative to implement a novel management pathway, which standardizes the process from start of care to final diagnosis, disposition and specific therapy of atraumatic abdominal pain patients in the ED. Therefore, a team of multidisciplinary experts has developed the „Abdominal Pain Unit“(APU) treatment process based on the Delphi method. The description of the details of this process will be published elsewhere.  The present study seeks to answer the following primary research questions:  Does the novel, standardized APU-process for patients with atraumatic abdominal pain in the ED lead to   1. a decrease in patient-reported acute pain at discharge from the ED? 2. an improvement of patient satisfaction at discharge from the ED, or / and 3. a shorter duration of treatment at the ED? |
|  | 6b | Explanation for choice of comparators | Patients in the control group will receive care as usual, which will be according the standard of care of each participating hospital, which might differ between study sites. |
| Objectives | 7 | Specific objectives or hypotheses | This study aims to improve German emergency department (ED) care for patients suffering from atraumatic abdominal pain. An application-supported pathway for the ED will be implemented, which supports quick, evidence-based, and standardized diagnosis and treatment plans for patients with atraumatic abdominal pain in the ED.  We hypothesize that the App-based APU-process will lead to:   1. Shorter duration of treatment at the ED while improving patient-reported outcomes (assessed as acute pain score or/and patient satisfaction) at discharge from the ED; or an 2. Improvement of patient-reported outcomes (assessed as acute pain score or/and patient satisfaction) at discharge from the ED while measuring a constant duration of treatment; or a 3. Shorter duration of treatment and unchanged patient-reported outcomes (assessed as acute pain score and patient satisfaction) at discharge from the ED.   All three scenarios will be interpreted as improvement of patients care by the new pathway. |
| Trial design | 8 | Description of trial design including type of trial (eg, parallel group, crossover, factorial, single group), allocation ratio, and framework (eg, superiority, equivalence, noninferiority, exploratory) | A mixed-methods multicenter cluster randomized controlled stepped wedge trial design will be applied. A total of 10 hospitals with EDs (expected n= 2.000 atraumatic abdominal pain patients) will consecutively (every 4 months) be enrolled into the intervention arm over a trial period of 24 months. |
| **Methods: Participants, interventions, and outcomes** | | | |
| Study setting | 9 | Description of study settings (eg, community clinic, academic hospital) and list of countries where data will be collected. Reference to where list of study sites can be obtained | As mentioned under 8), this study is a multicenter trial, in which of different size, specialization and location in Germany are included.  Campus Charité Mitte (CCM) Berlin  Campus Virchow-Klinikum (CVK) Berlin  Evangelische Elisabeth Klinik Berlin (Pilot- Hospital),  Helios Klinikum Berlin Buch Notfallzentrum  Uniklinikum Augsburg - Zentrale Notaufnahme  München Klinik Bogenhausen  Uniklinikum Jena - Zentrale Notaufnahme  Klinikum Bremen Nord - Interdisziplinäre Notaufnahme  Klinikum Wolfsburg - Zentrale Notaufnahme  Uniklinikum Greifswald - Zentrale Notaufnahme  Klinikum Fürth - Zentrale Notaufnahme |
| Eligibility criteria | 10 | Inclusion and exclusion criteria for participants. If applicable, eligibility criteria for study centers and individuals who will perform the interventions (eg, surgeons, psychotherapists) | Patients being 18 years or older presenting at the participating EDs with atraumatic abdominal pain will be screened for eligibility in the study. If patients have a legal representative, this representative has to agree to participation in the study. Exclusion criteria are traumatic causes of abdominal pain, (suspicion of) sepsis (quick Sequential (Sepsis-Related) Organ Failure Assessment: qSOFA score ≥ 2 [4]) or (suspicion of) shock (shock index ≥ 1), not being insured with a German SHI or insufficient knowledge of German language. |
| Interventions | 11a | Interventions for each group with sufficient detail to allow replication, including how and when they will be administered | Intervention group:  The App-supported APU-process (in the following and for clarity, the term APU-process will be used) starts with a patient who suffers from atraumatic abdominal pain presenting in the ED. By means of the APU-App, physicians will be supported in making a structured decision for the subsequent diagnostic and treatment process. Eventually, the APU-process ends with either the discharge of the patient with a sufficiently accurate diagnosis from the ED or the patient being admitted to another hospital unit for further treatment. Patients with a shock syndrome or sepsis leave the path for special intensive care early.  The first step in the APU-process includes a medical history, a medical examination, measurement of blood parameters, and pain management for patients with atraumatic abdominal pain.  In a second step, a re-evaluation will lead to a decision whether the patient will be discharged from the ED to ambulant care (i.e. in case of unsuspicious clinical findings) or if further diagnostic measures have to be taken. Thus, in a third step, the patient will receive a sonography, however, in the case of persistently unclear clinical findings, in a fourth step, either additional imaging methods will be used, such as computer tomography or magnetic resonance imaging, or a multi-disciplinary consultation and if necessary a patient observation for a few hours will be performed. Patients with worsening medical condition will leave the path for intensive care.  Control group: Care as usual in the ED. |
|  | 11b | Criteria for discontinuing or modifying allocated interventions for a given trial participant (eg, drug dose change in response to harms, participant request, or improving/worsening disease) | If a patient’s situation worsens (e.g. shock or sepsis), then the patient will immediately be admitted to intensive care. Patients will receive usual care, for instance, with regards to pain management.  See 11a. |
|  | 11c | Strategies to improve adherence to intervention protocols, and any procedures for monitoring adherence (eg, drug tablet return, laboratory tests) | All physicians participating in the study, as well as all study nurses will receive comprehensive APU schooling (by peers) as well as schooling in Good Clinical Practice. Also study nurses will receive comprehensive schooling about study specific data management tasks and in Good Clinical Practice. |
|  | 11d | Relevant concomitant care and interventions that are permitted or prohibited during the trial | Not applicable. |
| Outcomes | 12 | Primary, secondary, and other outcomes, including the specific measurement variable (eg, systolic blood pressure), analysis metric (eg, change from baseline, final value, time to event), method of aggregation (eg, median, proportion), and time point for each outcome. Explanation of the clinical relevance of chosen efficacy and harm outcomes is strongly recommended | Primary outcomes, except duration of treatment in the ED, are assessed at t0 (at discharge from the ED):   - Acute pain score (NRS, numeric rating scale: 0-10, [5]); - Duration of treatment in the ED (assessed in hospital records: time point of patient registration and documented time point when physician decides that the patient can leave the ED) - Patient satisfaction will be assessed with two items extracted from the Züricher Patientenzufriedenheit Fragebogen (ZUF-8, [6]).   Secondary outcomes:   - **Patient safety and quality of care** (e.g. quality of life: EUROHIS-QoL-8, [7]; mortality; course of treatment in the hospital. - **Process quality**: quantitative data of e.g. process times, such as time until diagnostic measures are available / diagnostic examination and qualitative data, such as potential success factors and pitfalls of the APU-process (e.g. feasibility in routine care, applicability in routine care). - **Cost- & Cost-effectiveness analysis** (e.g. costs of hospital stays, outpatient visits, medication, diagnoses and related costs) |
| Participant timeline | 13 | Time schedule of enrolment, interventions (including any run-ins and washouts), assessments, and visits for participants. A schematic diagram is highly recommended (see Figure) | Since the implementation of the APU-process will affect the entire ED, individual randomization of patients within centres is not feasible. Therefore, an alternative approach was chosen: the introduction of the process in the 10 study centres will take place in clusters of centres. As illustrated in Appendix 1, this stepped wedge design [8-10] involves a sequential, consecutive crossover of clusters (every 4 months) from the control arm to the intervention arm until all clusters are exposed. In this way, one can observe equal periods of control and intervention.  Data collection is set to a time period of 2 years, until all clusters (n=10) will have used the intervention for at least 4 months. |
| Sample size | 14 | Estimated number of participants needed to achieve study objectives and how it was determined, including clinical and statistical assumptions supporting any sample size calculations | An a-priori power calculation based on the primary endpoints, i.e. acute pain score at discharge from ED, duration of treatment in the ED, and patient satisfaction. Calculations were conducted with the nQuery Advisor 7.0. Calculations were corrected for the stepped wedge design with 10 centres and led to a total sample size of n= 1.700 patients needed for analysis. Considering potentially 15% study attrition rate the enrolment should aim for 2.000 patients (about n=200 patients per study arm). The significance level was set to α=0.05. |
| Recruitment | 15 | Strategies for achieving adequate participant enrolment to reach target sample size | Physicians working at the ED of participating study sites will be responsible for detecting and recruiting eligible patients presenting with atraumatic abdominal pain at the ED. Study nurses will support recruitment of patients and data collection.  Moreover, data collection will be regularly monitored internally by means of auditing and viewings of so-called screening logs at the study sites.  Reaching the targeted number of participants is assumed to be realistic as timely resources were considered in sample size calculations. For instance, we considered the number of patients presenting with atraumatic abdominal pain in each participating ED in previous years.  Also see 13 |
| **Methods: Assignment of interventions (for controlled trials)** | | | |
| Allocation: |  |  |  |
| Sequence generation | 16a | Method of generating the allocation sequence (eg, computer-generated random numbers), and list of any factors for stratification. To reduce predictability of a random sequence, details of any planned restriction (eg, blocking) should be provided in a separate document that is unavailable to those who enrol participants or assign interventions | Not applicable (see 8 & 15) |
| Allocation concealment mechanism | 16b | Mechanism of implementing the allocation sequence (eg, central telephone; sequentially numbered, opaque, sealed envelopes), describing any steps to conceal the sequence until interventions are assigned | Not applicable. |
| Implementation | 16c | Who will generate the allocation sequence, who will enrol participants, and who will assign participants to interventions | Also, see 15. |
| Blinding (masking) | 17a | Who will be blinded after assignment to interventions (eg, trial participants, care providers, outcome assessors, data analysts), and how | Not applicable. |
|  | 17b | If blinded, circumstances under which unblinding is permissible, and procedure for revealing a participant’s allocated intervention during the trial | Not applicable. |
| **Methods: Data collection, management, and analysis** | | | |
| Data collection methods | 18a | Plans for assessment and collection of outcome, baseline, and other trial data, including any related processes to promote data quality (eg, duplicate measurements, training of assessors) and a description of study instruments (eg, questionnaires, laboratory tests) along with their reliability and validity, if known. Reference to where data collection forms can be found, if not in the protocol | Recruitment of participants will be carried out in all 10 German hospital sites in the EDs by medical physicians. Study nurses will support data collection in the EDs (i.e., administering patient surveys and documentation).  Quantitative and qualitative data will be collected in five steps (i.e. modules) to facilitate cross verification of data and to enhance credibility of the results [11].  Module 1) Patients who present in the ED with atraumatic abdominal pain will be asked by physicians to participate in the APU study. Patients agree to study participation by signing the consent form.  At discharge from the ED (t0), study nurses will collect outcomes, i.e. acute pain scores (numerical rating scale, NRS 0-10), patient satisfaction (ZUF-8), quality of life (QoL; EUROHIS-QoL-8), and socio-economic data with tablets based questionnaire. At 30-day post-ED discharge (t1), study nurses either call patients or send them a link to access the online survey for the follow-up patient-reported measurements, such as patient’s acute pain scores and their quality of life (same as t0) or perform an in-hospital follow-up if the patient is still hospitalized.  Module 2) Study nurses will extract patient’s clinical parameters (e.g. vital parameters, pre-existing conditions, onset of pain, and procedures and results) and duration of treatment at the ED from hospital records to an electronic Case Report Form at t0 and t1. Moreover, at t1, re-admission data will be extracted.  Module 3) Routinely collected data from all patient suffering from atraumatic abdominal pain (identified by physicians), such as transport, timestamps, diagnosis, procedures, vital parameters, and blood parameters, as well as data from the potentially subsequent inpatient stay of all patients with atraumatic abdominal pain treated at the ED during the two-year study period will be extracted. Those data will be de facto anonymized. |
|  |  |  | Module 4) Module 4 consists of two different parts of data extraction, namely part a) and b):   1. Patients who consented to participate in the APU study and are also insured with the participating health insurance company, will be asked to agree to the provision of routinely collected data by the insurance company to the evaluating institute. These data include frequency and amount of health care resource consumption, health data, such as diagnosis and associated health care costs one year prior to trial until t1. These data will be economically investigated by the evaluating institute from the perspective of the health insurance company in terms of costs and cost-effectiveness (total costs related to primary outcomes) of the APU-process compared to controls over the whole study duration. In addition, an economic evaluation from the perspective of the hospital will be performed to investigate whether the usage of resources in the ED might have changed. For this analysis, data collected in Module 3 will be used (e.g., the number of ED procedures). 2. Moreover, the participating health insurance company will perform an internal evaluation of effects from data of all insured patients with atraumatic abdominal pain syndrome at the same duration (i.e. one year prior to trial until t1).   Module 5: Experts (n=20, i.e. physicians working with the APU-process in the EDs) and patients from the intervention group (n=25) enrolled in the APU study will be identified through purposive sampling. Interviews will be conducted at all locations. Prior to expert interviews, participatory patient observations will be conducted and documented as field notes which will subsequently be transferred into standardized observational protocols. Semi-structured interviews will be conducted via phone or face-to-face. Preceding the interviews, written informed consent will be given by participants |
|  | 18b | Plans to promote participant retention and complete follow-up, including list of any outcome data to be collected for participants who discontinue or deviate from intervention protocols | To ensure complete data for Module 1, study nurses will be in reach for participant’s questions during collection of patient-reported outcomes. For the 30-days follow-up (t1), participants will be contacted by phone, via email for the online link of the survey, or perform an in-hospital follow-up (in case participants are still hospitalized). In case participants cannot be reached, mortality data will be requested from the registration office.  Participants can stop their participation at any time, without giving a reason. Stopping their participation will not entail any disadvantages for participants.  Members of the project coordination and from the evaluating institutes will closely monitor participant recruitment and all contact with patient (e.g. for Follow-up) and must report all delays. |
| Data management | 19 | Plans for data entry, coding, security, and storage, including any related processes to promote data quality (eg, double data entry; range checks for data values). Reference to where details of data management procedures can be found, if not in the protocol | All measurement instruments for patient-reported outcomes data collection are tested for validity in prior scientific studies.  Primary data from module 2 will be extracted from the electronic documentation system of the hospital into electronic Case Report Forms. Pseudonymized IDs will allow subsequent data triangulation / linkage of data from different modules and different time points.  Data management will be performed with secuTrial®, a data management software. A trained data manager will monitor data collection and verification process. All data will be either pseudonymized (module 1, 2, 4a) or anonymized (module 3, 4b, 5).  Primary data, such as patient-reported outcomes and secondary data from hospital records or from the participating health insurance (Module 4a) will be pseudonymized. Primary data collected from patient observations and expert interviews as well as secondary data from the participating health insurance company (Module 4b) will be available as anonymized data. |
| Statistical methods | 20a | Statistical methods for analysing primary and secondary outcomes. Reference to where other details of the statistical analysis plan can be found, if not in the protocol | Descriptive and associated statistical analysis will be performed to provide summaries and analytical results for patient-reported outcomes, process measures, quality measures and costs. The three primary outcomes will be analyzed as planned for the RCT implementing adjustment methods necessary for data received in a stepped wedge study design. Generalized linear mixed models (GLMM) will be used to analyze primary outcomes as these allow also non-normal standard deviations and binary outcomes. Moreover, GLMM can compensate for differing cluster size (e.g. number of participants differs between study sites). The detailed SAP is currently prepared based on information about the data structure provided by the clinical team and the data managing team. In case of unequally distributed confounders (e.g., age, gender case mix) or potential risk factors (e.g., smoking or post-operative state of patient) in the data, sensitivity analysis will be performed.  Primary qualitative data collected in Module 5, consisting of field notes from participatory observations and the transcripts from semi-structured expert interviews will be analyzed inductively. |
|  | 20b | Methods for any additional analyses (eg, subgroup and adjusted analyses) | Not applicable.  In case of cluster effects, potential confounders (e.g., age and gender case mix) or distribution of potential risk factors (e.g., smoking or post-operative state of patient) will be investigated in additional sensitivity analyses. |
|  | 20c | Definition of analysis population relating to protocol non-adherence (eg, as randomized analysis), and any statistical methods to handle missing data (eg, multiple imputation) | For the primary outcomes, cases with missing primary outcomes (only applicable to the 2 patient-reported outcomes) will be distinguished into in-hospital death and other reason of lost-to-follow-up (LOF); multiple imputation (MI) of missing data will be based on variables selected by medical expertise (expectation).  Missing demographic and clinical data will also be handled with multiple imputation.  Corresponding analysis methods for MI data will be used accordingly. Always, complete case analysis and pooled results of MI data will be reported. |
| **Methods: Monitoring** | |  |  |
| Data monitoring | 21a | Composition of data monitoring committee (DMC); summary of its role and reporting structure; statement of whether it is independent from the sponsor and competing interests; and reference to where further details about its charter can be found, if not in the protocol. Alternatively, an explanation of why a DMC is not needed | The project data manager will perform data linkage and regular data checks (based on regular interim data exports) and data verification on the primary patient-reported outcomes and secondary data from hospital records performed by the statistician and inform the study monitor about data queries.  A health economics expert and member of this project, will perform cost analysis and cost-effectiveness evaluation from the perspective of the hospital with secondary data from hospital records and linked health insurance data. |
|  | 21b | Description of any interim analyses and stopping guidelines, including who will have access to these interim results and make the final decision to terminate the trial | No interim analysis are planned and no stopping rules apply. A pilot project at one study site will collect data of 50 randomly selected patients only to test data collecting systems (e.g. Tablet, App), and for verification and testing of technical systems and export processes (e.g. from the clinic system and the collected patient reported data). |
| Harms | 22 | Plans for collecting, assessing, reporting, and managing solicited and spontaneously reported adverse events and other unintended effects of trial interventions or trial conduct | Adverse events and other unintended effects of trial will be reported by the principal investigator within 3 working days.  In a pilot phase, preventive measures will be taken, to avoid:  Technical problems or confusion by physicians due to applying the new pathway to the patient with atraumatic abdominal pain, which subsequently could lead to delay of treatment. However, all physicians will receive schooling about the APU-App, which should prevent such delays in treatment. |
| Auditing | 23 | Frequency and procedures for auditing trial conduct, if any, and whether the process will be independent from investigators and the sponsor | The project will be implemented under the consortium leadership of the Emergency and Acute Medicine, Campus Mitte und Virchow Klinikum Charité. The scientific evaluation is led by the Institute of Medical Sociology and Rehabilitation Science and the Institute of Health Services Research in Emergency Medicine together with the Institute of Medical Biometrics and Clinical Epidemiology and the Institute of Social Medicine, Epidemiology and Health Economics of the Charité–Universitätsmedizin Berlin. TMF e.V. is responsible for data protection aspects, such as the creation and coordination of the data protection concept, as well as for aspects regarding medical device regulations.  Moreover, an external advisory board will monitor and regularly audit project processes. |
| Ethics and dissemination | |  |  |
| Research ethics approval | 24 | Plans for seeking research ethics committee/institutional review board (REC/IRB) approval | Ethical approval was obtained from Charité’s ethics committee (EA2/219/20) |
| Protocol amendments | 25 | Plans for communicating important protocol modifications (eg, changes to eligibility criteria, outcomes, analyses) to relevant parties (eg, investigators, REC/IRBs, trial participants, trial registries, journals, regulators) | Progress of the study, as well as all changes to the study protocol will be communicated to the funder (German Federal Joint Committee’s (G-BA) Innovation Funds) in regular interim reports. |
| Consent or assent | 26a | Who will obtain informed consent or assent from potential trial participants or authorised surrogates, and how (see Item 32) | Prior to informed consent, physicians will inform patients about the study and will then ask for informed consent to participate from patients.  Informed consent for expert interviews will be obtained prior to the interviews. |
|  | 26b | Additional consent provisions for collection and use of participant data and biological specimens in ancillary studies, if applicable | Not applicable |
| Confidentiality | 27 | How personal information about potential and enrolled participants will be collected, shared, and maintained in order to protect confidentiality before, during, and after the trial | Participants will give consent to the trial on paper by signing the informed consent form. Data will either be collected in a paper-pencil manner (patient-reported outcomes) or digitally, on tablets, as well as by means of data in the hospital records (i.e. KIS-system).  Informed consent forms of module 1, 2 and 4a will be stored locally at sites for 10 years in a steel cupboard according to official guidelines. A digital copy of these informed consent forms will be used to create a consent and identification management database at Charité. Only an authorized project member will have access. The data of the database will be archived after the end of the project and stored for 10 years.  Informed consent forms of module 5 will be stored for 10 years in a steel cupboard in the evaluating institute, according to official guidelines. |
| Declaration of interests | 28 | Financial and other competing interests for principal investigators for the overall trial and each study site | None to declare. |
| Access to data | 29 | Statement of who will have access to the final trial dataset, and disclosure of contractual agreements that limit such access for investigators | Only the principal investigator and project team members who are authorized will have access to trial data at location. |
| Ancillary and post-trial care | 30 | Provisions, if any, for ancillary and post-trial care, and for compensation to those who suffer harm from trial participation | Not applicable. |
| Dissemination policy | 31a | Plans for investigators and sponsor to communicate trial results to participants, healthcare professionals, the public, and other relevant groups (eg, via publication, reporting in results databases, or other data sharing arrangements), including any publication restrictions | Findings will be disseminated in publications in peer-reviewed journals, on conferences, as well as via a project Website (German language Website): <https://apu.charite.de> |
|  | 31b | Authorship eligibility guidelines and any intended use of professional writers | Not applicable. |
|  | 31c | Plans, if any, for granting public access to the full protocol, participant-level dataset, and statistical code | Not applicable. |
| Appendices | | | |
| Informed consent materials | 32 | Model consent form and other related documentation given to participants and authorized surrogates | See Appendix 2 (German language) |
| Biological specimens | 33 | Plans for collection, laboratory evaluation, and storage of biological specimens for genetic or molecular analysis in the current trial and for future use in ancillary studies, if applicable | None. |

*It is strongly recommended that this checklist be read in conjunction with the SPIRIT 2013 Explanation & Elaboration for important clarification on the items. Amendments to the protocol should be tracked and dated. The SPIRIT checklist is copyrighted by the SPIRIT Group under the Creative Commons “[Attribution-NonCommercial-NoDerivs 3.0 Unported](http://www.creativecommons.org/licenses/by-nc-nd/3.0/)” license.

**References:**

1. Mockel M, Searle J, Muller R, Slagman A, Storchmann H, Oestereich P, Wyrwich W, Ale-Abaei A, Vollert JO, Koch M *et al*: **Chief complaints in medical emergencies: do they relate to underlying disease and outcome? The Charité Emergency Medicine Study (CHARITEM)**. *European journal of emergency medicine : official journal of the European Society for Emergency Medicine* 2013, **20**(2):103-108.

2. Fagerström A, Paajanen P, Saarelainen H, Ahonen-Siirtola M, Ukkonen M, Miettinen P, Paajanen H: **Non-specific abdominal pain remains as the most common reason for acute abdomen: 26-year retrospective audit in one emergency unit**. *Scandinavian journal of gastroenterology* 2017, **52**(10):1072-1077.

3. Laurell H, Hansson L-E, Gunnarsson U: **Acute abdominal pain among elderly patients**. *Gerontology* 2006, **52**(6):339-344.

4. Singer M, Deutschman CS, Seymour CW, Shankar-Hari M, Annane D, Bauer M, Bellomo R, Bernard GR, Chiche J-D, Coopersmith CM: **The third international consensus definitions for sepsis and septic shock (Sepsis-3)**. *Jama* 2016, **315**(8):801-810.

5. Berthier F, Potel G, Leconte P, Touze M-D, Baron D: **Comparative study of methods of measuring acute pain intensity in an ED**. *The American journal of emergency medicine* 1998, **16**(2):132-136.

6. Kriz D, Nübling R, Steffanowski A, Wittmann WW, Schmidt J: **Patientenzufriedenheit in der stationären Rehabilitation: Psychometrische Reanalyse des ZUF-8 auf der Basis multizentrischer Stichproben verschiedener Indikation**. *Zeitschrift für medizinische Psychologie* 2008, **17**(2, 3):67-79.

7. Schmidt S, Mühlan H, Power M: **The EUROHIS-QOL 8-item index: psychometric results of a cross-cultural field study**. *European Journal of Public Health* 2005, **16**(4):420-428.

8. Brown CAL, Richard J: **The stepped wedge trial design: a systematic review**. *BMC Medical Research Methodology* 2006, **6**(1):54.

9. Hemminga K, Taljaard, Monica: **Sample size calculations for stepped wedge and cluster randomised trials: a unified approach**. *Journal of Clinical Epidemiology* 2016, **69**:137-146.

10. Köberlein-Neu J, Hoffmann F: **Das Stepped Wedge Design: Stufenlos regelbar?** *Zeitschrift für Evidenz, Fortbildung und Qualität im Gesundheitswesen* 2017, **126**:1-3.

11. Moon MD: **Triangulation: A Method to Increase Validity, Reliability, and Legitimation in Clinical Research**. *Journal of Emergency Nursing* 2019, **45**(1):103-105.

# Appendices

Appendix 1: Stepped Wedge Design


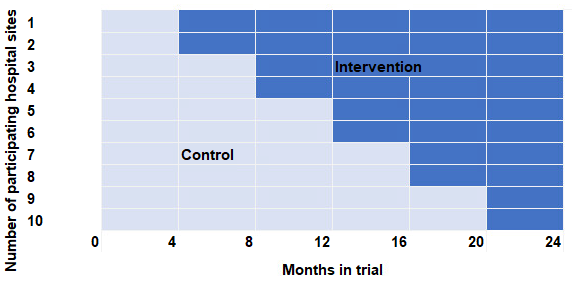


Appendix 2: Informed Consent form (German Language)

**Arbeitsbereich Notfall- und Akutmedizin**

Ärztlicher Leiter: Univ.-Prof. Dr. med. M. Möckel

**Wissenschaftliche Arbeitsgruppe „Abdominal Pain Unit“**


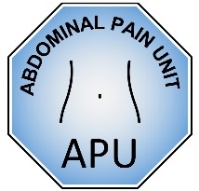

**Leiter des Prüfzentrums:**

Univ.-Prof. Dr. med. M. Möckel

Tel.: 030 / 450 553742

**Studienkoordinatorin:**

Britta Stier

Tel.: 030 / 450 631317

**Studienarzt:**

Charité | Campus Mitte | 10098 Berlin

Dr. med. L. Helbig

lukas.helbig@charite.de

**Einwilligungserklärung**

für die Teilnahme an der Studie: **Die Abdominal Pain Unit: Standardisierte strukturierte Versorgung von Patient*innen mit atraumatischen Bauchschmerzen in der Notaufnahme (APU)**

Hiermit erkläre ich

dass ich durch Herrn/Frau ...................................................................................................

*(Name des Studienarztes/ der Studienärztin/der Studienkoordinatorin/dem Studienkoordinator)*

mündlich und schriftlich (über die Studieninformation) über das Wesen, die Bedeutung und die Risiken der wissenschaftlichen Untersuchungen im Rahmen der o.g. Studie informiert wurde und ausreichend Gelegenheit hatte, meine Fragen mit dem Studienarzt/ der Studienärztin/ der Studienkoordinatorin/ dem Studienkoordinator zu klären.

Mir ist bekannt, dass ich das Recht habe, meine Einwilligung jederzeit ohne Angabe von Gründen und ohne nachteilige Folgen für mich zurückzuziehen und von der Studienleitung jederzeit Auskunft, Berichtigung, Sperrung und Löschung meiner Daten verlangen oder einen Widerspruch gegen die Datenverarbeitung einlegen kann. Hierzu wende ich mich an die Studienleitung (zentrale Anlaufstelle), die meine Daten re-identifizieren kann. Außerdem kann ich Beschwerde bei einer Datenschutzbehörde einlegen.

Ich habe eine Kopie der schriftlichen Studieninformation und der Einwilligungserklärung erhalten.

**Ich erkläre, dass ich freiwillig bereit bin, an der wissenschaftlichen Studie teilzunehmen.**

**Die Teilnahme beinhaltet das Ausfüllen eines Fragebogens im Rahmen der Vorstellung in der Notaufnahme sowie eine erneute Befragung nach 30 Tagen per Telefon oder per Onlinefragebogen. Je nach Zeitpunkt des Einschlusses wird nach dem App-gestützten APU-Behandlungspfad oder nach gängiger klinischer Praxis behandelt.**

1. **Ich bestätige, dass ich die Teilnehmerinformation „Die Abdominal Pain Unit: Standardisierte strukturierte Versorgung von Patient*innen mit atraumatischen Bauchschmerzen in der Notaufnahme (APU)“ gelesen und verstanden habe. Die Studie wurde mir erklärt und ich hatte die Gelegenheit, Fragen zu stellen.**

APU - Einwilligungserklärung Version 2.0 vom 18.03.2021 Seite **2** von **1**

1. **Ich bin damit einverstanden, dass meine für den Zweck der o.g. Studie nötigen personenbezogenen Daten durch den Studienarzt / die Studienärztin / den Studienkoordinator / die Studienkoordinatorin erhoben, pseudonymisiert aufgezeichnet und verarbeitet werden dürfen, auch auf elektronischen Datenträgern;**
2. **dass die während meines Krankenhausaufenthalts anfallenden Daten aus den Behandlungsakten und dem Krankenhausinformationssystem (Blutdruck, Puls, Sauerstoffsättigung, Körpertemperatur, Wachheit, Vorerkrankungen, Schmerzbeginn, Schmerzende, Schmerzskala, begleitende Symptome, Medikamentenanamnese, Behandlungsende, Blutparameter, Urinparameter, Vorerkrankungen, Untersuchungen und Ergebnisse, Diagnosen, Prozeduren, Operationen und den Krankenhausverlauf (Stationen, Intensivstation, Länge des Aufenthaltes, Komplikationen, Mortalität, Diagnoseschlüssel, Abrechnungsziffern)) durch die Studienmitarbeiter*innen extrahiert und verarbeitet werden dürfen;**
3. **dass meine Studiendaten an die am APU-Projekt beteiligten Institute der Charité übermittelt und verarbeitet werden dürfen;**
4. **dass die Studienergebnisse in anonymer Form, die keinen Rückschluss auf meine Person zulässt, veröffentlicht werden dürfen.**
5. **Ich bin damit einverstanden, dass meine Daten bis zu zwei Jahre nach Förderende abschließend analysiert und am Ende der Studie für 10 Jahre gemäß der „guten wissenschaftlichen Praxis“ aufbewahrt werden. Danach werden sie vernichtet.**
6. **Ich bin damit einverstanden, dass die Studienergebnisse in anonymer Form veröffentlicht werden und dass bei wissenschaftlichen Datenarchiven die Datensätze ohne Pseudonym oder mit einem neuen Pseudonym hinterlegt werden dürfen.**
7. **Ich erkläre mich damit einverstanden, dass autorisierte und zur Verschwiegenheit verpflichtete Beauftragte des Studienleiters sowie die zuständigen inländischen und ausländischen Überwachungsbehörden in meine beim Prüfarzt vorhandenen personenbezogenen Daten, insbesondere meine Gesundheitsdaten, soweit dies für die Überprüfung der ordnungsgemäßen Durchführung der Studie notwendig ist, Einsicht nehmen. Für diese Maßnahme entbinde ich den Prüfarzt von der ärztlichen Schweigepflicht.**
8. **Ich erkläre mich damit einverstanden, dass die Mitarbeiter des APU-Projektes – soweit es für die Studiendurchführung von APU erforderlich ist – meine personenbezogenen Daten einschließlich Gesundheitsdaten bei Ärzten, Pflegepersonen sowie bei Bediensteten von Krankenhäusern erheben und für diese Zwecke verwenden. Insoweit gestatte ich der Studienleitung sowie von dieser beauftragten Personen Einsicht in meine Patientenakte, in Untersuchungsbefunde sowie Arzt- und Krankenhausberichte. Ich entbinde die genannten Personen und Mitarbeiter der genannten Einrichtungen von ihrer Schweigepflicht und gestatte die Übermittlung von o.g. Dokumenten für die Erfassung des Versorgungsverlaufes an die Mitarbeiter des APU-Projektes soweit sie für die Zwecke der Studie erforderlich sind.**
9. **Mir ist bekannt, dass ich diese Erklärungen über die Entbindung von der Schweigepflicht jederzeit mit Wirkung für die Zukunft widerrufen kann.**
10. **Ich möchte Informationen zum Fortschritt und den Ergebnissen des Projekts per E‑Mail in Form eines Newsletters alle 3 Monate erhalten und stimme Verarbeitung meiner E-Mail-Adresse zu diesem Zwecke zu**

- **ja**
- **nein**

APU - Einwilligungserklärung Version 2.0 vom 18.03.2021 Seite **2** von **2**

1. **Für Versicherte der Techniker Krankenkasse:**

**Ich bin damit einverstanden, dass für den Zweck o.g. Studie meine verfügbaren Abrechnungsdaten nach §75 SGB V an die am Projekt beteiligten Institute der Charité als Empfänger**

- - **übermittelt werden dürfen. Diese Daten umfassen sowohl die ambulanten wie stationären Leistungsbereiche ein Jahr vor, sowie einen Zeitraum von 30 Tagen nach dem Erstkontakt in der Notaufnahme.**
  - **Ich bin nicht damit einverstanden, dass für den Zweck o.g. Studie meine verfügbaren Abrechnungsdaten nach §75 SGB V an die evaluierenden Institute übermittelt werden dürfen.**

**Notizen zum Inhalt des Aufklärungsgesprächs:**

APU - Einwilligungserklärung Version 2.0 vom 18.03.2021 Seite **3** von **3**

**Ich gebe hiermit meine Einwilligung an der o.g. Studie teilzunehmen und willige in die studienbezogene Verarbeitung personenbezogener Daten ein und bestätige dies durch meine Unterschrift**.

__________________________________________________________________________

(Name und Vorname des Patienten)

__________________________________ _________________________________

(Geburtsdatum) (Telefonnummer)

__________________________________ _________________________________

(TK-Versichertennummer - falls zutreffend) (E-Mail-Adresse)

__________________________________ _________________________________

(Ort und Datum) (Unterschrift des Patienten)

Teilnehmercode (Pseudonym, wird von der Studienleitung eingetragen): ………………...........

**Hiermit erkläre ich, den/die Teilnehmer/in über Wesen, Bedeutung und Risiken der o.g. Studie mündlich und schriftlich aufgeklärt, alle Fragen beantwortet und ihm/ihr eine Kopie der Studieninformation und der Einwilligungserklärung übergeben habe.**

**……………………… ………..……………………………………………………………….**

Ort und Datum Name Unterschrift des aufklärenden Studienarztes / der aufklärenden Studienärztin

**Einwilligung gesetzliche*r Betreuer*in:**

Ich____________________________(Name gesetzliche*r Betreuer*in), als gesetzlicher Betreuer*in von ____________________________________(Name Studienteilnehmer*in), geboren_________________(Geburtsdatum Studienteilnehmer*in) gebe hiermit meine Einwilligung, dass Herr/Frau __________________________________ an der o.g. Studie teilnimmt und willige in die studienbezogene Verarbeitung personenbezogener Daten ein und bestätige dies durch meine Unterschrift.

APU - Einwilligungserklärung Version 2.0 vom 18.03.2021 Seite **4** von **4**

__________________________________ _________________________________ (Name und Vorname gesetzlich*er Betreuer*in) (Geburtsdatum)

__________________________________ _________________________________ (Ort und Datum) (Unterschrift gesetzlich*er Betreuer*in)

Teilnehmercode (Pseudonym, wird von der Studienleitung eingetragen): ………………...........

APU - Einwilligungserklärung Version 2.0 vom 18.03.2021 Seite **5** von **5**
