# Supplementary material for: The Abdominal Pain Unit (APU). Study protocol of a standardized and structured care pathway for patients with atraumatic abdominal pain in the emergency department: A stepped wedged cluster randomized controlled trial
Source: PLoS One. 2022 Aug 24;17(8):e0273115. doi: 10.1371/journal.pone.0273115 (PMC9401147; doi:10.1371/journal.pone.0273115)
Supplement: S1 Protocol — (PDF) [file pone.0273115.s002.pdf]

Antrag auf Beratung durch die Ethikkommission zur Durchführung eines medizinisch-wissenschaftlichen Vorhabens, welches weder die klinische Prüfung eines Arzneimittels noch Medizinproduktes beinhaltet

1. Titel der Studie	Die **A**bdominal **P**ain **U**nit: Standardisierte strukturierte Versorgung von Patient*innen mit *Atraumatischen Bauchschmerzen* in der Notaufnahme (APU)
2. Ethikkommissions -Antragsnummer	**EA2/219/20**
3. Entscheidungen anderer Ethikkommissionen in derselben Sache	/
4. Gegenstand der Studie und ihre Ziele; Angabe der Hypothesen, getrennt in Haupt- und Sekundärhypothesen sowie der klinischen Parameter (primäre und sekundäre Endpunkte), anhand derer die Hypothesen geprüft werden sollen	Patient*innen mit Bauchschmerzen ohne Trauma sollen schneller, sicherer und zielführender versorgt werden. Dies gelingt, indem ein neuer Versorgungsprozess implementiert wird, der Symptom- und Prozess- und nicht Diagnose-bezogen strukturiert ist. Dieser Prozess soll Software gestützt anhand einer App umgesetzt werden. Die Implementierung eines neuen strukturierten, symptombasierten Prozesses ist notwendig, da Patient*innen in der Notaufnahme sich primär mit einem Symptom und nicht mit einer bereits bestehenden Diagnose vorstellen. In dieser Weise wird die Indikationsqualität der durchgeführten Diagnostik verbessert und Patient*innen können schneller und effizienter behandelt werden.<br><br><u>Hypothesen:</u><br>Die standardisierte Versorgung von Patient*innen mit atraumatischen Bauchschmerzen in der Notaufnahme anhand des neuen Abdominal Pain Unit (APU)-Prozesses bewirkt<br>Haupthypothese:<br>1) eine Verkürzung der Behandlungszeit bei gleichzeitiger Verbesserung der patientenbezogenen Endpunkte (Patientenzufriedenheit und Schmerzintensität) bei Verlassen der Notaufnahme<br>Sekundärhypothesen:<br>2) eine Verbesserung der patientenbezogenen Endpunkte (Patientenzufriedenheit und Schmerzintensität) bei Verlassen der Notaufnahme bei gleichbleibender Behandlungszeit oder<br>3) eine Verkürzung der Behandlungszeit und unveränderte patientenbezogene Endpunkte (Patientenzufriedenheit und Schmerzintensität) bei Verlassen der Notaufnahme.<br><br><u>Primäre Endpunkte:</u><br>Akuter Schmerz bei Verlassen der Notaufnahme, Patientenzufriedenheit, Dauer der Notaufnahmeversorgung.<br><br><u>Sekundäre Endpunkte:</u><br>Prozessevaluation, Qualitätsverbesserung, Gesundheitsökonomie
5. Erläuterung der Bedeutung der Studie	Die Ergebnisse der Studie haben eine große Relevanz für die Versorgung von Patient*innen mit atraumatischen Bauchschmerzen in der Notaufnahme.<br>Atraumatische Bauchschmerzen haben eine hohe Prävalenz (zwischen 2015 und 2017 zeigten ca.

	18,5% aller Patient*innen, die in den Notaufnahmen der Charite CCM+CVK vorstellig wurden, das Leitsymptom Bauchschmerz) und eine hohe Mortalität (intrahospitale Mortalität ca. 5%). Gleichzeitig können sehr verschiedene Ursachen für Bauchschmerzen bestehen, die von schwerwiegenden Notfällen (z.B. Mesenterialinfarkt) bis zu weniger zeitkritisch behandlungsbedürftigen Diagnosen (z.B. psychosomatische Ursachen) reichen.<br>Angesichts der demographischen Situation (Zunahme älterer Bevölkerungsgruppen, Zunahme pflegebedürftiger, multimorbider und teilweise dementer Gruppen) ist damit zu rechnen, dass die Anzahl an Patient*innen mit unspezifischer Bauchschmerzsymptomatik weiterhin zunehmen wird. Gleichzeitig besteht aus verschiedenen, insbesondere auch ökonomischen Gründen, eine deutliche zeitliche Limitierung der Behandlungszeit pro Patient*in und eine teilweise deutliche Überlastung des Personals in den Notaufnahmen. Dies führt, neben hoher Patientenunzufriedenheit, zu der Gefahr, dass schwerwiegende Diagnosen übersehen werden (Unterversorgung) oder das es zur Überdiagnostik und damit verbunden Nachteilen (z.B. Strahlenbelastung) kommt.<br><br>Es besteht somit der Bedarf für einen evidenzbasierten, standardisierten Versorgungsprozess für Patient*innen mit atraumatischen Bauchschmerz.<br>Für Patient*innen mit akuten Brustschmerz und Patienten mit V.a. Schlaganfall haben sich bereits erfolgreich solche Versorgungsprozesse im Rahmen von „Chest Pain Units“ und „Stroke Units“ etabliert.<br>Bezüglich des App-unterstützten Versorgungsprozesses der „Abdominal Pain Unit“ fehlen bisher randomisierte Studien zur Validierung des Konzeptes.<br>Die Studie hat das Ziel diese Forschungslücke zu schließen.
6. Welche der folgenden Bestimmungen finden Anwendung<br>a) Medizinproduktegesetz gemäß § 23b MPG - Ausnahme der klin. Prüfung<br>b) Strahlenschutzgesetz und Strahlenschutzverordnung<br>c) Gendiagnostikgesetz<br>d) Datenschutzgesetze:<br>- Konkrete Angabe des durch die verantwortliche Stelle zu erfüllenden Datenschutzgesetzes (für die Charité =<br>-EU-Datenschutzgrundverordnung (DSGVO), Berliner Datenschutzgesetz - BlnDSG).<br>- Ggf. entsprechend des Teilnehmerkreises zusätzlich zu beachtende Landesdaten-schutzgesetze oder BDSG.	Erläuterung: nach Prüfung durch multiple Instanzen, welche im Folgenden aufgezählt sind ist die App kein Medizinprodukt und das Medizinproduktegesetz kommt nicht zur Anwendung.<br><br>1.) Clinical Trial Office, BIH, Dr. Uwe Behrens,<br>2.) Leitung der Ethikkommission Lageso Berlin, Frau Antje Kettner-Ottilie<br>3.) Referat für Medizinprodukte, Lageso, Inspektor Medizinprodukte Dr. Matthias Merx,<br><br>d) Es kommen das Berliner- und das Bundes-Datenschutzgesetz zur Anwendung. Ob ggf. weitere Datenschutzgesetze (Landesdatenschutzgesetz) Anwendung finden, wird durch die Datenschutzbeauftragte der Charité geprüft.
7. Ggf.: Bezeichnung und Charakterisierung der Prüfprodukte	Es kommt kein physisches Prüfprodukt zur Anwendung.

	Bei dem APU-Behandlungsprozess handelt es sich um einen strukturierten, standardisierten Pfad zur Behandlung von atraumatischen Bauchschmerzen. Die einzelnen Abschnitte des Behandlungsprozesses und ihre Darstellung in der unterstützenden APP sollen hier beispielhaft erläutert werden. Anschließend gehen wir spezifisch auf die Rolle der APP in dem Prozess ein. Zum besseren Verständnis finden Sie im Anhang die erweiterte Ereignisgesteuerte Prozesskette (eEPK) des APU-Behandlungsprozesses (Anhang 4), die als Grundlage unseres Projektes im Delphi-Verfahren von Expert*innen erarbeitet wurde.<br><u>Ablauf des APU-Prozesses</u><br>Am Anfang des Behandlungsprozesses steht die Standard Operation Procedure (SOP, Checkliste) „Klinische Einschätzung Schock/Sepsis“ (*Feld 3* in der eEPK, Anhang 7), welche das medizinische Personal dabei unterstützen soll, vital gefährdete Patienten frühzeitig zu erkennen.<br>Die SOP beinhaltet dabei unter anderem folgende Fragen an den behandelnden Arzt: Haben Sie Blutdruck, Herzfrequenz, Sauerstoffsättigung, Wachheit geprüft? Ist der Patient kreislaufstabil Ja? Nein? Es werden außerdem Hilfestellungen zur Berechnung der Wachheit (Glascow Coma Scale) oder zur Berechnung klinischer Scores (in diesem Fall qSOFA-Score zur Einschätzung ob Sepsis/Schock vorliegt) gegeben.<br>Sobald ein Schock nach der entsprechenden SOP ausgeschlossen ist, erfolgt die Durchführung der Basismaßnahmen. Diese beinhalten die Anamnese und körperliche Untersuchung *(Feld 10*), Basislabordiagnostik (*Feld 14*), sowie die Durchführung eines adäquaten Schmerzmanagements (*Feld 18*). Die hierfür hinterlegten SOPs sollen dem behandelnden Arzt Hilfestellung für ein mögliches, effizientes Vorgehen liefern.<br>Alle genannten SOPs sind Teil des Behandlungsprozesses und werden über die APP informativ angezeigt.<br>Im Anschluss an die Basismaßnahmen erfolgt die SOP „Klinische Einschätzung“ (*Feld 23*) des Patienten, welche wir hier exemplarisch in Anhang darstellen. In dieser SOP wird die Frage gestellt, ob nun eine gesicherte oder weiterhin unklare Diagnose besteht. Der Arzt muss nun, anhand der Informationen, die er selbst in der Anamnese oder in der körperlichen Untersuchung, erhoben hat, sowie der Laborbefunde, die er aus dem lokalen Krankenhausinformationssystem (KIS) entnimmt (welches nicht in die APP übertragen wird) entscheiden, ob hier zum Zeitpunkt der erneuten klinischen Einschätzung, eine „gesicherte Diagnose“ oder noch immer eine „unklare Diagnose“ oder gar ein „Schock“ vorliegt.<br>Beispielhafte ist eine SOP in Anhang 7dargestellt.<br>Sollte nun eine gesicherte Diagnose vorliegen, führt der Behandlungsprozess, wie auch ohne Studie, zum Behandlungsende und damit zur SOP „Spezifisches Management“ (Feld 23).

	Sollte keine gesicherte Diagnose vorliegen, wird dem Arzt die Sonografie als weitere Diagnostik vorgeschlagen und daraus folgende mögliche weitere Schritte des Behandlungsprozesses (Observation, radiologische Bildgebung, Konsil). Auf diese genannten weiteren Schritte gehen wir an dieser Stelle nicht detailliert ein. Sie unterscheiden sich aber im Aufbau und Prinzip nicht von den vorher aufgeführten SOPs.<br>Am Ende des Prozesses steht, wie oben bereits erwähnt, die SOP „Spezifisches Management“, anhand derer überprüft werden kann, ob alle für das Ende der Behandlung wichtigen Informationen eingeholt wurden und die Voraussetzungen zur Entlassung/Verlegung/Intervention/Operation vorhanden sind.<br><br><u>Rolle der APP in dem APU-Behandlungsprozess</u><br>In dem hier generell und anhand der SOP „Klinische Einschätzung“ (s. Anhang 7) detaillierter beschriebenen Behandlungsprozess unterstützt die APP die ärztlichen Aufgaben wie folgt:<br>• Anzeigen der Standard Operating Procederes<br>• Dokumentation von Zeitstempeln<br>• Dokumentation von gesetzten „Häkchen“ in Checklisten<br>• Berechnen klinischer Scores (wie auch im Klinikinformationssystem üblich)<br>Die genannten SOPs enthalten explizit *keine verbindlichen* Handlungsaufforderungen, die der Arzt anwenden muss. In der SOP „Anamnese“ bekommt er z.B. typische Symptome, die mit Bauchschmerzen einhergehen, tabellarisch dargestellt. Bei der SOP „Labor Bauchschmerz“ kann er sich durchlesen, welche Laborparameter, nicht vergessen werden sollten. Er muss diese jedoch nicht auswählen. Damit steht die der APP unterstützte Pfad nicht in Konkurrenz zu den üblichen Verfahrensanweisungen der Kliniken.<br>In den einzelnen SOPs existieren keine „Pflichtfelder“. Der Anwender kann stets frei entscheiden welche Aspekte des APU-Behandlungsprozesses wahrgenommen oder gegebenenfalls übersprungen werden. Über die Dokumentationsfunktion der App kann in diesem Zusammenhang die Prozesstreue erfasst werden.<br>Der APU-*Behandlungsprozess*, welcher in unserer Studie evaluiert werden soll, dient dem Zweck der „Erkennung, Verhütung, Überwachung, Behandlung oder Linderung von Krankheiten“.<br>Die unterstützende APP selbst hat allerdings keinen Einfluss auf den Behandlungsprozess als solchen. Sie zeigt lediglich dessen Inhalt an und erlaubt die Dokumentation, ob der Anwender die entsprechenden Informationen zur Kenntnis genommen hat. Sie dient ausdrücklich nicht dem Zweck der *geräteseitigen* Erkennung, Verhütung, Überwachung, Behandlung oder Linderung von Krankheiten. Sie verwendet keine künstliche Intelligenz und trifft zu keinem Zeitpunkt Entscheidungen für den Anwender und schlägt diese auch nicht vor. Die Entscheidung und Interpretation

	von Befunden obliegt weiterhin allein dem behandelnden ärztlichen Personal. Allerdings wird sichergestellt, dass die nach dem „state of the art“ notwendigen Informationen und Befunde vorliegen.<br><br>Zusammenfassend handelt es sich beim APU-Prozess um ein Behandlungsprozedere, welches Software-gestützt über eine Applikation („App“) auf stationären (z.B. Desktop-Pcs) und/oder mobilen Endgeräten (z.B. Tablets) Anwendung findet.<br><br>Hierbei wird nach den gängigen Behandlungsleitlinien behandelt und der APU-Prozess gibt lediglich eine standardisierte Struktur des Behandlungspfades vor. Ziel ist hierbei keine diagnostischen und therapeutischen Schritte zu übersehen, ähnlich einer Checkliste. Genauso ist aber auch Ziel, keine unnötigen therapeutischen Schritte oder Diagnostiken durchzuführen. Es werden hierbei keine neuen diagnostischen oder therapeutischen Maßnahmen implementiert. Es wird lediglich der Behandlungspfad an sich, der eine standardisierte Struktur der Behandlung von atraumatischen Bauchschmerzen vorgibt, implementiert. Die Behandlung wird damit für das ärztliche Personal strukturierter und sicherer. Die hierbei verwendete App dient als Instrument, auf welcher der APU Prozess dargestellt ist.
8. wesentliche Ergebnisse der vorklinischen Tests oder Gründe für die Nichtdurchführung derselben	Entfällt
9. Wesentlicher Inhalt und Ergebnisse der vorangegangenen Studien/Anwendungen der in der Studie zu prüfenden Produkte	Uns ist keine Studie bekannt, die einen evidenzbasierten, standardisierten Versorgungsprozess für Patienten mit atraumatischen Bauchschmerz etabliert und validiert.<br>Zwar wurde beispielsweise von Trentzsch und Kollegen 2011 ein klinischer Algorithmus zur Diagnostik und Therapie akuter Abdominalschmerzen in der Notaufnahme entwickelt. (Anlage 1 (Trentzsch, Werner et al. 2011)) Dieser wurde allerdings nie prospektiv, klinisch und randomisiert erprobt.<br><br>Literatur:<br>Trentzsch, H., et al. (2011). "Der akute Abdominalschmerz in der Notfallambulanz – ein klinischer Algorithmus für den erwachsenen Patienten." Zentralbl Chir 136(02): 118-128.
10. Beschreibung der vorgesehenen Maßnahmen/Untersuchungsmethoden und eventuelle Abweichungen von den in der med. Praxis üblichen Maßnahmen/Untersuchungen (was ist „Routine“, was wird davon abweichend in der Studie gemacht?)<br><br>Kommen validierte Fragebögen studienbedingt zum Einsatz, geben Sie bitte die Bezeichnung der Fragebögen an und wo diese publiziert sind (Referenzen).<br>Nicht validierte Fragebögen bitte als Anlage beifügen.	Es handelt sich um eine multizentrische, randomisierte Interventionsstudie.<br><br><u>Untersuchungsmethoden:</u><br>Kern der Studie ist ein multizentrisches, Stepped-Wedge Cluster Randomized Controlled Trial (cRCT). Im Anschluss an die Pilotphase in einer teilnehmenden Klinik werden die zehn weiteren teilnehmenden Studienkliniken randomisiert fünf Gruppen mit fünf verschiedenen Startperioden zugeordnet. Diese starten konsekutiv, jeweils zu Beginn des Folgequartals, mit der Umsetzung der neuen Abdominal Pain Unit (APU). Mit dem Start der

	Umsetzung der neuen Versorgungsform werden die Patienten für die Interventionsgruppe rekrutiert. In dem Zeitraum von Projektbeginn bis zum jeweiligen Start der Intervention erfolgt die Rekrutierung der Kontrollgruppenpatient*innen, welche die bisherige Regelversorgung erhalten.<br><br><u>Standardvorgehen während der Kontrollphase:</u><br>Die Versorgung in der Kontrollgruppe erfolgt weiter anhand der jeweils vorherrschenden hausinternen Standards.<br>Diese sind nicht immer evidenzbasiert und unterscheiden sich teilweise deutlich von Krankenhaus zu Krankenhaus. Weiterhin ist die Genauigkeit der Umsetzung dieser Handlungsempfehlungen häufig von der Erfahrung und Intuition des behandelnden Arztes/der behandelnden Ärztin abhängig.<br><br><u>Abweichung vom Standardvorgehen in der Interventionsphase:</u><br>Im APU-Prozesses werden gängige Behandlungsleitlinien angewandt, ohne deren Inhalt im Kern zu verändern.<br>Patient*innen mit Bauchschmerzen ohne Trauma sollen schneller, sicherer und zielführender versorgt werden. Dies gelingt, indem ein neuer Versorgungsprozess implementiert wird, der Symptom- und Prozess- und nicht Diagnose-bezogen strukturiert ist. Die Implementierung eines neuen strukturierten, symptombasierten Prozesses ist notwendig, da Patient*innen in der Notaufnahme sich primär mit einem Symptom und nicht mit einer bereits bestehenden Diagnose vorstellen. In dieser Weise wird die Indikationsqualität der durchgeführten Diagnostik verbessert und Patient*innen können schneller und effizienter behandelt werden.<br>Dieser Versorgungsprozess soll Software gestützt anhand einer App dargestellt werden. Hierbei dient die App als zeitgemäßes Mittel zur Darstellung des Prozesses und gleichzeitig als Dokumentationssoftware im Kontext der Anwendung des neuen Behandlungspfades. Die App selbst hat weder einen Einfluss auf den Versorgungsprozess noch schreibt sie dem Arzt, der die App nutzt vor, wie die Behandlung / Therapie zu erfolgen hat.<br>Sie zeigt lediglich dessen Inhalt an und dokumentiert, ob der/die Anwender*in die entsprechenden Informationen zur Kenntnis genommen hat. Sie dient ausdrücklich *nicht* dem Zweck der geräteseitigen Erkennung, Verhütung, Überwachung, Behandlung oder Linderung von Krankheiten. Sie stellt keine künstliche Intelligenz dar und trifft zu keinem Zeitpunkt Entscheidungen für den/die Anwender*in. Die Entscheidung obliegt weiterhin dem behandelnden ärztlichen Personal. Somit handelt es sich bei der App um eine Dokumentationssoftware im Kontext der Prozessoptimierung durch den APU-Versorgungsprozess.<br>Schwerpunkt der Studie soll der neue Versorgungsprozess sein. Das Ziel unserer Studie ist nicht die Zulassung der App als Medizinprodukt,

	sondern die Evaluation und ggf. Zulassung des neuen APU-Behandlungsprozesses.<br><br>Die App erhält Informationen durch das behandelnde ärztliche Personal.<br>Zu den Informationen zählen unter anderem ein Patientenidentifikator, der Name, das Alter des/der Patient*in.<br><br>Der neue Prozess hat das Ziel, unnötigen therapeutische oder diagnostische Schritte zu vermeiden bzw. durch die standardisierte Struktur zu verhindern, dass keine wichtigen Befunde übersehen werden oder dringend notwenigen Maßnahmen ausgelassen werden. Der Schwerpunkt liegt hier in dem Aufzeigen, der verschiedenen diagnostischen und therapeutischen Maßnahmen ohne eine abschließende Empfehlung abzugeben. Es geht darum, den Behandlungsprozess ähnlich einer Checkliste abzuarbeiten und die Behandlung zu optimieren.<br>Der Prozess ist bisher als Event-Prozess-Kette (Event process chain = EPC) dargestellt. Die schematische Darstellung findet sich im Anhang 4<br><br>Das Versorgungsprozedere ist gegenüber dem „Routinevorgehen“ somit stärker standardisiert und objektiviert.<br><br>Die Evaluation der Studie verfolgt einen Mixed-Methods-Ansatz. Es werden quantitativ und qualitativ erhobene Primärdaten mit Sekundärdaten des Krankenhausinformationssystems sowie Abrechnungsdaten der beteiligten Krankenkassen trianguliert. Weiterhin sind Experteninterviews geplant.<br><br>Die Evaluation erfolgt geordnet nach Modulen zu verschiedenen Zeitpunkten:<br>• Modul 1 (Patientenberichtete Primärdaten): Kontroll- und Interventionsgruppe werden bei der Entlassung aus der Notaufnahme via Tablet-Computer (bei Bedarf als Paper-Pencil) befragt (Erhebung t0). Wenn Patient*innen nicht in der Notaufnahme befragt werden können, (z.B. aufgrund einer Verlegung in den OP) erfolgt bis zu 72 Stunden nach dem initialen Kontakt in der Notaufnahme eine Nacherhebung auf der Behandlungsstation auf welche der/die Patient*in verlegt wurde. Nach 30 Tagen erfolgt ein telefonisches Follow-up oder eine Onlinebefragung (t1). Die Patient*innen werden zur Erinnerung an das Follow-up telefonisch oder per E-Mail vorab kontaktiert. Die Analysen umfassen die aktuelle Schmerzintensität (numerische Ratingskala (NRS)), Zufriedenheit mit der eigenen Gesundheit und Lebensqualität (Items entnommen aus EUROHIS-QOL-8; Anlage 2: (Schmidt, Mühlan et al. 2005)), soziodemografische Daten, sowie die Patientenzufriedenheit ((ZUF-8) Anlage 3

	(Schmidt, Wittmann et al. 2002)) und Lebenszufriedenheit ((Kurzskala Lebenszufriedenheit-1 (L-1) Anlage 4 (Beierlein et al. 2014.))<br>Siehe Anlage t0 und t1 Fragebogen<br>• Modul 2 (Primärdaten der Versorgung): Für jede*n Patienten*in erfolgt die Erfassung klinischer Parameter (z.B. Vitalparameter, Vorerkrankungen, Schmerzbeginn, begleitende Symptome, Medikamentenanamnese) sowie die Dauer des Aufenthaltes in der Notaufnahme. Die Erhebung erfolgt ohne Beteiligung der Patient*innen nach deren Entlassung aus der Notaufnahme.<br>• Modul 3 (Sekundärdaten der Klinik): Für Patient*innen mit Bauchschmerzen im Studienzeitraum erfolgt die Extraktion der für sie verfügbaren Daten aus dem Krankenhausinformationssystem (KIS). Die KIS-Daten umfassen zum Beispiel Blutparameter, diagnostische Untersuchungen und Ergebnisse, Diagnosen, Prozeduren, Operationen und den Krankenhausverlauf (Stationen, Intensivstation, Länge des Aufenthaltes, Komplikationen, Mortalität, DRGs, Abrechnungsziffern) der Patient*innen. Die Erhebung erfolgt durch Studienmitarbeiter*innen ohne Beteiligung der Patient*innen nach deren Entlassung aus der Notaufnahme.<br>• Modul 4 (Sekundärdaten der Krankenkasse): Mit der Erklärung ihrer Teilnahme an der Studie willigt die Patient*in ein, dass ihre Krankenkasse (aktuell TK, BKK) die verfügbaren Abrechnungsdaten nach §75 SGB V an das evaluierende Institut übermitteln darf. Die übermittelten Daten umfassen sowohl die ambulanten wie stationären Leistungsbereiche ein Jahr vor Indexaufenthalt sowie einen Zeitraum von 30 Tagen nach dem Erstkontakt. Die Daten können im Längsschnitt analysiert werden. Wichtige Parameter sind die zeitlichen Verläufe in Form von Krankheitsverläufen, der Mortalität und der in Anspruch genommenen Leistungen. Weiterhin werden in den Daten der beteiligten gesetzlichen Krankenversicherungen alle während des Studienzeitraums behandelten Patient*innen identifiziert und intern (bei der beteiligten Krankenkasse) bezüglich der vor und nach Krankenhausaufenthalt entstandenen Kosten analysiert. Die Erhebung erfolgt ohne Beteiligung der Patient*innen.<br>• Modul 5 (Experteninterviews, teilnehmende Beobachtung): Im Rahmen der teilnehmenden Beobachtung werden je 5 Patient*innen in 5 Kliniken nach der Etablierung der Abdominal-Pain-Units über den Zeitraum ihres Notaufnahmeaufenthaltes begleitet (n=25). Die Beobachtung startet mit dem Behandlungsstart und Endet mit der Entlassung aus der Notaufnahme. Die Beobachtungen, welche auch das gesprochene Wort beinhalten, werden systematisch in Form von Protokollen

	dokumentiert. Weiter werden je Klinik 2 Expert*inneninterviews nach Einführung der Abdominal Pain Unit innerhalb der Projektlaufzeit geführt (total n= 20). Die Expert*inneninterviews erfolgen als leitfadengestützte Interviews, werden tonbandprotokolliert und transkribiert.<br><br>Literatur:<br>Schmidt, J., et al. (2002). Fragebogen zur Messung derPpatientenzufriedenheit. Diagnostische verfahren in der psychotherapie. Göttingen: Hogrefe.<br><br>Schmidt, S., et al. (2005). "The EUROHIS-QOL 8-item index: psychometric results of a cross-cultural field study." European Journal of Public Health 16(4): 420-428.<br><br>Beierlein et al. (2014). Eine Single-Item-Skala zur Erfassung der Allgemeinen Lebenszufriedenheit: Die Kurzskala Lebenszufriedenheit-1 (L-1). GESIS-Working Papers 2014 | 33. GESIS: Köln.
11. Bewertung und Abwägung der vorhersehbaren Risiken und Nachteile der Studienteilnahme gegenüber dem erwarteten Nutzen für die Studienteilnehmer und zukünftig erkrankte Personen (Nutzen-Risiko-Abwägung)	
a. zu prüfender medizinischer Nutzen für die Studienteilnehmer (individueller Nutzen für den einzelnen Patienten)	Es wird davon ausgegangen, dass durch die Anwendung des APU-Prozesses die Indikationsqualität der durchgeführten Diagnostik verbessert und Patient*innen schneller und effizienter behandelt werden können.<br>Der individuelle Nutzen für den/die einzelne*n Patient*in würde sich in einer Verkürzung der in der Notaufnahme zu verbringenden Zeit, einer schnelleren und adäquateren Schmerztherapie, sowie in einer erhöhten Zufriedenheit mit der Behandlung zeigen.<br><br>Modul 5: Der individuelle Nutzen für die Studienteilnehmer*innen (Expert*innen) an den Expert*inneninterviews, würde sich in einer möglichen Reflektion und Verbesserung der Anwendung und Arbeitsprozesse im Zusammenhang mit der APU-App auswirken. Bezüglich der teilnehmenden Patientenbeobachtungen wird kein unmittelbarer individueller Nutzen zu erwarten sein.
b. zu prüfender medizinischer Nutzen für zukünftig erkrankte Personen (Gruppennutzen)	Für zukünftig erkrankte Personen würde sich der Nutzen ebenfalls in einer schnelleren und effizienteren Versorgung von Patient*innen mit atraumatischen Bauchschmerzen wiederspiegeln. Es ist anzunehmen, dass sich die genannten positiven Effekte mit zunehmender Etablierung des APU-Prozesses noch weiter verstärken und somit Wartezeiten und Schmerzlevel abnehmen und Patientenzufriedenheit zunehmen werden.<br>Darüber hinaus schaffen die Ergebnisse der Studie eine wissenschaftliche Basis für eine weitere

	Optimierung der Versorgung von Bauchschmerzpatient*innen in der Notaufnahme.
c. **Risiken** und Belastungen für die Studienteilnehmer (alle im Einzelnen auflisten)	Die Anwendung des APU-Prozesses selbst stellt kein zusätzliches Risiko dar, da der APU-Prozess lediglich gängige Behandlungsleitlinien in eine standardisierte Struktur bringt ohne diese im Kern zu verändern oder neue, bisher klinische nicht etablierte Verfahren zur Anwendung zu bringen.<br>Diese Behandlungsleitlinien umfassen beispielsweise:<br>- Klinische Untersuchung<br>- Anamnese<br>- Laboruntersuchungen<br>- Bildgebende Verfahren (z.B. Sonografie, CT)<br>- Fachärztliche Konsile<br>- Schmerzmanagement<br>- Re-Evaluation und Therapie von Schock, Sepsis zu vorgegebenen Zeitpunkten<br><br>Es werden somit ausschließlich Verfahren angewendet, die bereits in der klinischen Routine etabliert sind.<br><br>Potentiell besteht das Risiko, dass es im Rahmen des APU-Prozesses zu technischen Problemen bei der Umsetzung des Prozesses oder zu Verwirrung beim anwendenden Personal kommt und sich Behandlungsentscheidungen somit verzögern. Um die App möglichst anwenderfreundlich zu gestalten und solche Risiken zu vermeiden wird die App in einer Pilotphase umfangreich im klinischen Setting getestet und die Anwendung optimiert.<br><br>Ansonsten besteht durch die Teilnahme an der Studie für die Teilnehmer kein unmittelbares Risiko und es ist kein Schaden zu erwarten.
12. Maßnahmen zur Risikobeherrschung	Um die oben genannten Risiken so gering wie möglich zu halten, ist geplant die APU-Applikation („App“) schon vor dem ersten Einsatz am Patienten ausgiebig zu testen, die Interoperabilität der App mit verschiedenen Geräten sicherzustellen und das teilnehmende Personal ausgiebig in der Anwendung des APU-Prozesses inklusive der Arbeit mit der App zu schulen. Die App wird weiterhin im Rahmen einer Pilotphase in einer Klinik getestet und weiter optimiert.
13. Abbruchkriterien	Falls die Studienteilnahme für Patient*innen oder Expert*innen eine unzumutbare Belastung darstellt, soll die Teilnahme abgebrochen werden.<br>Patient*innen werden darauf hingewiesen, dass sie jederzeit auch ohne Angabe von Gründen ihre Einwilligung zurückziehen und die Studie abbrechen können.
14. Anzahl, Alter und Geschlecht der betroffenen Personen	<u>Studienpopulation:</u><br>Alle volljährigen Patient*innen aller Geschlechter, die im angegebenen Zeitraum mit den unter Punkt 16 angegebenen Ein- und Ausschlusskriterien vorstellig werden, geschätzt 2.000 Patient*innen.

	Modul 5: Für die leitfadengestützten Interviews werden n=20 volljährige Expert*innen aller Geschlechter befragt.<br>Für die teilnehmenden Beobachtungen werden n=25 volljährige Patient*innen aller Geschlechter beobachtet.
15. Biometrische Planung mit Angabe der statistischen Methodik, einschließlich der Begründung der Fallzahl.<br>Angabe des/der Statistikers/Statistikerin (sofern Beratung durch das Institut für Biometrie der Charité erfolgt, muss eine Unterschrift eingefügt werden)	Begründung der gewählten Fallzahlen/Stichprobengrößen (Power-Berechnung)<br>Bei der Evaluation einer neuen Versorgungsform mit einem breiten Fokus auf die Verbesserung der Patientenversorgung gibt es keine einzelne Forschungshypothese, die geprüft werden soll, sondern eine Reihe von kombinierten Endpunkten (Prozesszeiten, patientenbezogene Endpunkte). Eine klassische Stichprobenberechnung wie in einer konfirmatorischen Wirksamkeitsstudie ist daher nicht möglich. Weiterhin ist die erreichbare Zahl von Patient*innen durch die Zentren und den dabei zu erwartenden Anteil von Notfallpatient*innen mit atraumatischem Bauchschmerz begrenzt. Die Begründung des Stichprobenumfangs basiert daher auf Machbarkeitsüberlegungen und dementsprechend wurde eine Power-Analyse für relevante Endpunkte der Evaluationsmodule 1 und 2 durchgeführt. Es wird insgesamt mit einer erreichbaren Fallzahl von 2.000 Patient*innen gerechnet. Wir erwarten bei konservativer Schätzung ein Loss to Follow-up von 15%, da der Verlegungszeitpunkt bzw. der Zeitpunkt der ambulanten Entlassung nicht immer vorhersehbar ist und die Erfassung der Endpunkte ggf. bei notfallmäßiger Verlegung nicht erfolgen kann. D.h. die zu erwartende Zahl auswertbarer Patient*innen reduziert sich auf mindestens n=1.700. Zur Korrektur für das Stepped-Wedge Design nach Hemming (Anlage 4: (Hemming and Taljaard 2016) wurden folgende Annahmen getroffen: es gibt zufällige Cluster-Effekte, feste Zeiteffekte, aber keine Interaktionen zwischen Cluster und Zeit. Die Intracluster-Korrelationskoeffizienten (ICC) liegen in humanen Studien üblicherweise zwischen 0.01 und 0.02 (Anlage 5: (Killip, Mahfoud et al. 2004). Bei Wahl der konservativen Variante eines ICC von 0.02, der auf diesem Intervall zum höchsten SWD-Korrekturfaktor führt, erhalten wir einen Korrekturfaktor von 2.71. Die Anwendung dieses Korrekturfaktors resultiert in einer effektiven, einem RCT-Design entsprechenden Gesamtfallzahl von n=627, also n=313 pro Behandlungsarm. Für die Powerabschätzungen wird das Signifikanzniveau für 3 parallele Tests nach Bonferroni auf $\alpha^*=0.05/3=0.0167$ adjustiert. Für die oben genannten, primären Endpunkte unter der jeweils angenommenen Effektstärke δ und α^* ergäben sich folgende Fallzahlen für ein RCT: 1) für $\delta=0.341$ in einem 2-seitigen t-Test werden n=183 pro Gruppe, 2) für $\delta=0.0863$ in einem Chi^2-Test werden n=105 pro Gruppe, und 3) für $\delta=0.499$ in einem 2-seitigen t-Test werden n=86 pro Gruppe benötigt. Die Powerberechnungen führen unter Prüfung der 3 oben

	definierten Testhypothesen zu dem Ergebnis, dass die Studie für alle 3 Endpunkte mit einem RCT-Äquivalent von n=313 Patient*innen pro Behandlungsarm ausreichend gepowert ist. Konkret könnte man für die angenommenen Effektstärken mit der erreichbaren, für das SWD korrigierten Fallzahl n=313 pro Behandlungsarm eine Power von 1) 96%, 2) 99% und 3) 99% erreichen. Speziell für die „Patient reported outcomes“ der Endpunkte 2 und 3 würden die angenommenen Fallzahlen und damit erreichbare Power die Adjustierung der Auswertungen für potentielle Confounder wie Alter und Geschlecht nicht von vornherein ausschließen. Die Fallzahlkalkulationen und Powerabschätzungen wurden mit nQuery Advisor 7.0 durchgeführt.<br><br>Statistikerin:<br>Dr. Dörte Huscher, Institut für Biometrie und Klinische Epidemiologie, Charité – Universitätsmedizin Berlin<br><br>Literatur:<br>Hemming, K. and M. Taljaard (2016). "Sample size calculations for stepped wedge and cluster randomised trials: a unified approach." Journal of Clinical Epidemiology 69: 137-146.<br><br>Killip, S., et al. (2004). "What is an intracluster correlation coefficient? Crucial concepts for primary care researchers." Ann Fam Med 2(3): 204-208.
16.<br>a. Darlegung und ggf. Erläuterung der **Ein- und Ausschlusskriterien**	Einschlusskriterien:<br>Volljährige Patient*innen mit atraumatischen Bauchschmerzen<br>Einwilligungsfähigkeit (selbst oder durch Betreuer)<br><br>Modul 5: Für leitfadengestützte Interviews werden Mitarbeiter*innen (z.B. Ärzt*innen der jeweiligen Notaufnahme befragt, die die neue APU-App anwenden.<br>Für die teilnehmenden Beobachtungen werden vereinzelte teilnehmende Patient*innen beobachtet.<br><br>Ausschlusskriterien:<br>Offensichtliches Trauma oder Unfall als Ursache der Bauchschmerzen<br>Vorliegen eines Schocks oder einer Sepsis (qSOFA ≥ 2) bei Aufnahme in die Notaufnahme<br><br>Modul 5: Leitfadengestützte Interviews: Expert*innen, die die APU-App nicht anwenden. Teilnehmende Beobachtungen: Keine.
b. **Studieninformation** (wer diese mündlich und schriftlich erteilt und Angabe, wie viel Zeit zwischen Aufklärung und Einwilligung verbleibt (schriftliche Information als Anlage)	Aufgrund der Heterogenität der Behandlungsprozesse in den Notaufnahmen kann die Bedenkzeit nicht quantifiziert werden. Potentielle Studieninteressierte werden aus diesem Grund zunächst nach der Ersteinschätzung in der Triage durch das Studienteam mündlich und schriftlich über die Studie vorinformiert. Die Bedenkzeit besteht bis zum Kontakt mit der/dem behandelnden Ärztin/Arzt/Studienarzt/Studienärztin. Die/der behandelnde Ärztin/Arzt/Studienarzt/Studienärztin

	informiert den/die Patient*in erneut und gibt dem/die Patient*in bis zum Behandlungsstart weitere Bedenkzeit. Die Studienteilnehmer*innen haben ausreichend Zeit, Fragen zu stellen. Eine längere Bedenkzeit kann nicht eingeräumt werden, da die Intervention an die Behandlung in der Notaufnahme gekoppelt ist und die Studie keinen unmittelbaren invasiven Charakter hat. Die Patient*innen werden über Risiken aufgeklärt (siehe Punkt 11c).<br><br>Modul 5: Expert*innen werden schriftlich mit einem Informationsschreiben über die Teilnahme an den Expert*inneninterviews aufgeklärt (z.B. per E-Mail oder in Schulungen) (siehe Anlage X). Die Zeit zwischen der Aufklärung und Einwilligung beträgt mindestens 24 Stunden.
c. **Einwilligungserklärung** (schriftliche Form als Anlage)	Für die Studie wird eine Einwilligungserklärung verwendet. Die Teilnehmer*innen erklären darin die Freiwilligkeit und ihr Einverständnis zur Teilnahme an der Studie (s. Anlage A)<br>Für die Expert*inneninterviews in Modul 5 wird eine separate Einwilligungserklärung verwendet (siehe Anlage X).
d. Ggf. **Information und Einwilligung des gesetzlichen Vertreters** (ggf. auch Beschreibung des Verfahrens zur Einrichtung einer gerichtlichen Betreuung)	Bei Patient*Innen mit gesetzlichem/-er Vertreter*in wird diese*r aufgeklärt und ein gesonderter Abschnitt in der Einwilligungserklärung für die Einwilligung verwendet. Die gesetzlichen Vertreter der Teilnehmer*innen erklären darin die Freiwilligkeit und ihr Einverständnis zur Teilnahme an der Studie (s. Anlage A)
17. Maßnahmen zur Gewinnung von Studienteilnehmern (Aushang?, Zeitungsannoncen? Etc.)	Die Identifikation von Studienteilnehmer*innen erfolgt in der Notaufnahme im Rahmen der pflegerischen Erstversorgung. Im Zeitraum von Projektbeginn bis zum jeweiligen Start der Intervention erfolgt die Rekrutierung der Kontrollgruppenpatient*innen, welche die bisherige Regelversorgung erhalten. Mit der Umsetzung der neuen Versorgungsform werden Patient*innen für die Interventionsgruppe rekrutiert. Die Identifikation erfolgt anhand des führenden Symptoms bei Vorstellung in der Notaufnahme (Bauchschmerz). Weitere Maßnahmen sind nicht geplant.<br><br>Modul 5: Die Rekrutierung von Expert*innen für die leitfadengestützten Interviews erfolgt während der Schulungen der Mitarbeiter / Anwender der APU-App bzw. per E-Mail als Einladungsschreiben zur Studienteilnahme mit Studieninformationen (z.B.: Informationen über die Zielsetzung, den Hintergrund, datenschutzrechtliche Regelungen der Studie, sowie eine Erläuterung zum Verfahren der Interviews).<br>Für die teilnehmenden Beobachtungen des Behandlungsprozesses von Patient*innen, siehe die Identifikation von Patient*innen in der Notaufnahme.

18. Ggf.: Grund für die Einbeziehung und Darlegung des therapeutischen Nutzens für Personen, die minderjährig und/oder nicht einwilligungsfähig sind.	An der Studie sollen möglichst alle Patient*innen mit atraumatischen Bauschmerz teilnehmen können, um eine repräsentative Stichprobe untersuchen zu können, die in Analysen in Subgruppen extrapoliert werden können. Bei Patient*Innen mit gesetzlichem Vertreter/gesetzlicher Vertreterin wird diese*r aufgeklärt und ein gesonderter Abschnitt in der Einwilligungserklärung für die Einwilligung verwendet. Es wird zuvor eine Kopie der Betreuungsvollmacht eingeholt.
19. Beziehung zwischen Studienteilnehmer und Studienarzt/-ärztin (Ist der Studienarzt zugleich der behandelnde Arzt?)	Die Studienärzt*innen können auch behandelnde Ärzt*innen der Studienteilnehmer*innen sein. Die Aufnahme in die Studie erfolgt nach Identifikation im Rahmen der pflegerischen Erstversorgung in der Notaufnahme durch den Studienarzt/die Studienärztin.
20. Erklärung zur Einbeziehung möglicherweise vom Sponsor abhängiger Personen	Es werden keine vom Sponsor abhängigen Personen in die Studie einbezogen.
21. Maßnahmen, die eine Feststellung zulassen, ob ein Studienteilnehmer an mehreren Studien zugleich oder vor Ablauf einer in der vorangegangenen Studie festgelegten Frist teilnimmt.<br>Ist die Teilnahme an mehreren Studien möglich?	Die Teilnahme an einer weiteren Studie ist möglich, sofern hierdurch keine Beeinträchtigung der zu untersuchenden Endpunkte erwartet wird.
22. Ggf.: Honorierung bzw. Kostenerstattung der Studienteilnehmer (Höhe, wofür soll gezahlt werden?)	Für die Teilnahme an den geplanten Studien erhalten Patient*innen kein Honorar. Es entstehen für die Patient*innen keine Kosten.
23. Ggf.: Plan für die Weiterbehandlung und medizinische Betreuung der betroffenen Personen nach dem Ende der Studie	Teilnehmende und nicht-teilnehmende Personen werden entsprechend der gültigen Leitlinien behandelt. Je nach Schweregrad erfolgt eine ambulante Weiterbehandlung oder eine stationäre Aufnahme der Patient*innen. Eine medizinische Betreuung/Behandlung ist auch nach Studienende innerhalb der Strukturen der beteiligten Kliniken gesichert.
24. Ggf.: Versicherung der Studienteilnehmer (Versicherungsbestätigung und Versicherungsbedingungen, Versicherer, Versicherungsumfang, Versicherungsdauer)	Aus unserer Sicht ist eine zusätzliche Versicherung der Studienteilnehmer*innen nicht notwendig. Die Haftpflichtversicherung der teilnehmenden Kliniken wird als ausreichend angesehen.
25. Dokumentationsverfahren:<br>- Ggf. Verweis auf CRF-Bögen<br>-Angabe der zu erfassenden Daten<br>- Probenumgang<br>- Aufbewahrung / Archivierung (inkl. Fristen)<br>- Zugang zu den Daten und Proben	Die Datenerfassung und die Auswahl der zu verwendeten Instrumente orientiert sich an internationalen Standards und wird im multidisziplinären Prozess entwickelt. Die finalen Datenerfassungsbögen werden nachgereicht.<br>Die Primärdatenerhebung erfolgt dezentral in 10 Kliniken für 2.000 Patient*innen. Die Primärdaten sollen in elektronischen Case Report Forms erfasst und zentral zusammengeführt werden. Die Zusammenführung erfolgt über die Dateneingabe und –managementsoftware SecuTrail. Für die Entwicklung von Datenerhebungsinstrumenten, die elektronische Umsetzung, die Pseudonymisierung, Datenzusammenführung, -extraktion, das Datenmonitoring, die Datenbereinigung und –plausibilisierung ist ein*e die wissenschaftliche Dokumentar*in geplant. Es werden einerseits die

	beschriebenen evaluationsspezifischen Daten zu Notaufnahme- bzw. Klinikaufenthalt erhoben (Primärdaten), wie auch Sekundärdaten verarbeitet. Sekundärdaten werden sowohl aus den Krankenhausinformationssystemen, wie auch von den beteiligten Krankenkassen zur Verfügung gestellt. Im Rahmen des Projektes wird eine zentrale, relationale Datenbank aufgebaut, in welcher Primär- und Sekundärdaten pseudonymisiert vorliegen werden. Die wissenschaftlichen Mitarbeiter*innen der beteiligten evaluierenden Institute, sowie der Biometriker*in des Projektes sind an allen Phase der Datenerfassung, -verarbeitung und –auswertung beteiligt. Qualitätssicherungsmaßnahmen beinhalten die Schulung von Mitarbeiter*innen in der Datenerfassung und die technische Unterstützung zur Implementierung der elektronischen Datenerfassung vor Ort in den beteiligten Notaufnahmen, Monitoring der Dateneingaben und –qualität vor Ort und zentral.<br><br>Modul 5:<br>Für die leitfadengestützten Interviews der Studienärzt*innen werden pro Studienzentrum zwei Studienärzt*innen befragt (n=20), die die APU-App anwenden. Es werden lediglich die reinen Einwilligungsdokumente zur Interviewteilnahme erfasst. Mittels einer Rekrutierung der Studienärzt*innen für die leitfadengestützten Interviews während der Anwenderschulungen für die APU-App bzw. per E-Mail als Einladungsschreiben zur Studienteilnahme mit Studieninformationen (z.B.: Informationen über die Zielsetzung, den Hintergrund, datenschutzrechtliche Regelungen der Studie, sowie eine Erläuterung zum Verfahren der Interviews) wird die E-Mailadresse der teilnahmebereiten Ärzt*innen verwendet. Das Interview wird als Audiodatei aufgezeichnet. Dabei werden keine personenidentifizierenden Daten der Ärzt*innen (oder von Patient*innen durch Interviewantworten) erhoben. Die Audiodateien werden datenschutzkonform an das Transkriptionsunternehmen übermittelt, dort transkribiert und wieder an das Institut für Medizinische Soziologie zurückübermittelt. Die Interviewtranskripte werden anonymisiert und zugangsbeschränkt auf den Servern der Charité gespeichert. Die Audiodateien der Interviews werden nach Erhalt der Interviewtranskripte an das Institut für Medizinische Soziologie bzw. nach Abschluss des Transkriptionsprozesses bei dem beauftragten Transkriptionsunternehmen gelöscht. Für die Durchführung der Transkription der Audiodateien wird eine Vereinbarung zur Auftragsverarbeitung zwischen Transkriptionsunternehmen und Charité gemäß Art. 28 DSGVO geschlossen. Der Zugang zu diesen Daten ist nur der/dem zuständigen Mitarbeiter*in im APU-Projekt erlaubt.<br><br>Für die teilnehmenden Beobachtungen werden n=25 Patient*innen während der Notaufnahmebehandlung beobachtet. Alle Daten werden anonym durch Studienmitarbeiter*innen vom Institut für Medizinische Soziologie erhoben, d.h. es werden keine

	personenbezogenen Daten in die Beobachtungskladden eingetragen, sondern die Anwendung der neuen Versorgungsform im „real-life“ Setting, sowie mögliche beeinflussende Kontextfaktoren (z.B. Notaufnahmeabläufe, Kommunikationsstrukturen, Patienten, Personal, etc.) beobachtet und protokolliert. Die anonymen Protokolle werden im Institut für Medizinische Soziologie aufbewahrt und analysiert.<br><br>Nur die Leiter*innen der Studie und der Studienzentren sowie Personal, welches mit dem Datenmanagement beauftragt wird, haben Zugriff auf die Studiendaten.<br>Die Studiendaten werden bis zu 2 Jahren nach dem Förderende der Studie analysiert.<br>Die Studiendaten werden anschließend 10 Jahre aufbewahrt und dann gelöscht. Ein entsprechendes Datenschutzkonzept wird mit der behördlichen Datenschutzbeauftragten der Charité und weiteren zuständigen Stellen erarbeitet.
26. Ggf.: Beschreibung, wie der Gesundheitszustand gesunder betroffener Personen dokumentiert werden soll	Nicht zutreffend.
27. Ggf.: Methoden, unerwünschte Ereignisse festzustellen, zu dokumentieren und mitzuteilen (wann, von wem und wie ?)	Unerwünschte Ereignisse werden im Rahmen des Indexaufenthaltes und des telefonischen Follow-up erfasst. Alle unerwünschten Ereignisse werden dem Studienleiter binnen 3 Werktagen gemeldet. Weitere Meldungen erfolgen bei Bedarf.
28. Vorgehen zum Schutz der Geheimhaltung der gespeicherten Daten, Dokumente und ggf. Proben, Darlegung der Pseudonymisierung oder Anonymisierung der Daten und Proben von Studienteilnehmern (Initialen und Geburtsdatum als Codierungsschema sind nicht zulässig!)<br>- Beschreibung der Trennung von Krankenakten, Studiendokumentation und Zuordnung der personenbezogenen Daten<br><br>- Nennung der Zugriffsrechte einschließlich des Zugangs zu Teilnehmeridentifikations-listen während und nach der Studiendurch-führung<br>- Detaillierte Angabe der Verfahren für die Übertragung, Verschlüsselung, Einschränkung der Verarbeitung (Sperrung) und Löschung (einschließlich Angabe der ggf. verwendeten Netzstruktur und verwendete Server).<br>-ggf. Zugang zu identifizierenden Daten für gesetzlich berechtigte Prüfer (Dritte) zur zweckgebundenen Einsichtnahme in die dafür erforderlichen Akten.	Den Studienteilnehmer*innen wird ein automatisiert generiertes Pseudonym zugeordnet.<br>Die Teilnehmeridentifikationsliste wird in den Räumen der jeweiligen Studienteams verschlossen aufbewahrt. Zugriff auf diese Unterlagen haben ausschließlich an der Studie beteiligtes Personal und die Studienleiter*innen vor Ort. Nach der Studiendurchführung wird die Teilnehmeridentifikationsliste bei dem Studienleiter*innen vor Ort in einem verschließbaren Stahlschrank für die gesetzlich vorgeschriebene Zeit von 10 Jahren aufbewahrt.<br><br>Modul 5:<br>Expert*inneninerviews sind pseudonymisiert, da keine personenbezogenen Daten erhoben werden. Nach Löschung der Audiofiles sind die Transkripte anonym. Teilnehmende Beobachtungen werden anonym erhoben.<br><br>Die Studiendaten (Expert*innen- & Patient*innendaten) werden NICHT an Dritte weitergeleitet.
29. Erklärung zur Einhaltung des Datenschutzes<br>- Zusicherung, dass alle über den Studien-teilnehmer erhobenen und gespeicherten	Alle über die Studienteilnehmer*innen/ Expert*innen erhobenen und gespeicherten Daten werden vertraulich (Datengeheimnis und ärztliche Schweigepflicht) behandelt.

Daten vertraulich (Datengeheimnis und ärztliche Schweigepflicht) behandelt werden.<br>- Zusicherung, dass die identifizierenden Daten nur dem Studienleiter oder von ihm beauftragten Mitarbeitern zugänglich sind.<br>- Angabe der Maßnahmen zur Sicherstellung der Vertraulichkeit<br>- Maßnahmen zur datenschutzgerechten Übermittlung von Daten, die für Dritte keinen Personenbezug herstellen lassen.<br>- Angaben zu Auskunfts-, Widerrufs-, Berichtigungs- und Löschmöglichkeiten,<br>- Maßnahmen zur Sicherstellung der Rechte der Teilnehmer.<br>- Falls Übermittlungen ins Nicht-EU-Ausland vorgesehen sind: Maßnahmen zur Einhaltung des Datenschutzes (z.B. Vorliegen eines Angemessenheitsbeschlusses der EU-Kommission oder explizite Einwilligung der Studienteilnehmer in solche Übermittlungen	Die identifizierenden Daten sind nur den Studienleiter*innen und den von ihnen beauftragten Mitarbeiter*innen zugänglich.<br><br>Für alle erhobenen Studiendaten besteht das Recht auf Auskunft. Dies bedeutet, dass die/der Teilnehmer/in zu jeder Zeit Anspruch auf Auskunft und Berichtigung bzw. Löschung der Studiendaten zu seiner/ihrer Person hat.
30. Namen und Anschriften der Einrichtungen, die als Studienzentrum oder Studienlabor in die Studie eingebunden sind, sowie der Studienleiter und der Studienärzte<br>- Angabe beteiligter externer Dienstleister mit Angabe der Datenzugriffsmöglichkeit	Studienzentrum<br>Charité Universitätsmedizin Berlin<br>Arbeitsbereich Notfall- und Akutmedizin CVK, CCM<br>Augustenburger Platz 1<br>13353 Berlin<br><br>Leiter der Prüfung und verantwortlicher Studienarzt:<br>Prof. Dr. Martin Möckel<br>030 450 553203<br>martin.moeckel@charite.de<br><br>Stellvertretende Leiterin der Prüfung und verantwortliche Epidemiologin:<br>Prof. Dr. Anna Slagman<br>030 450 565659<br>anna.slagman@charite.de<br><br>Studienärzt*innen:<br>PD Dr. Undine Gerlach-Runge<br>030 450 531000<br>undine.gerlach@charite.de<br><br>Britta Stier<br>030 450 631317<br>britta.stier@charite.de<br><br>Dr. Lukas Helbig<br>030 450 631356<br>lukas.helbig@charite.de<br><br>Myrto Bolanaki<br>030 450 665653<br>myrto.bolanaki@charite.de<br><br>Dorothee Riedlinger<br>030 450 631316<br>dorothee.riedlinger@charite.de

	Rebecca Resendiz Cantu<br>030 450 631338<br>rebecca.resendiz@charite.de<br><br>Weitere Studienzentren und Studienleiter*innen:<br><br>Kooperation:_<br>Dr. med. Maik Kilian<br>Evangelische Elisabeth Klinik Berlin<br>Lützowstraße 26, 10785 Berlin<br>info.elisabeth@pgdiakonie.de<br>030 2506-1<br><br>Prof. Christian Wrede<br>Helios Klinikum Berlin Buch Notfallzentrum<br>Schwanebecker Chaussee 50, 13125Berlin<br>christian.wrede@helios-gesundheit.de<br>030 94 01-54700<br><br>Dr. med. Markus Wehler<br>Uniklinikum Augsburg - Zentrale Notaufnahme<br>Stenglinstr. 2, 86156 Augsburg<br>sekretariat.noa@uk-augsburg.de<br>0821 400-3876<br><br>Prof. Christoph Dodt<br>München Klinik Bogenhausen<br>Englschalkinger Straße 77, 81925 München<br>christoph.dodt@klinikum-muenchen.de<br>089 9270-3269<br><br>Prof. Wilhelm Behringer<br>Uniklinikum Jena - Zentrale Notaufnahme<br>Am Klinikum 1, 07747 Jena<br>wilhelm.behringer@med.uni-jena.de<br>03641 9-322001<br><br>Dr. med. Frank Wösten<br>Klinikum Bremen Nord, Interdisziplinäre Notaufnahme<br>Hammersbecker Straße 228, 28755 Bremen<br>frank.woesten@gesundheitnord.de<br>0421 6606-1950<br><br>Dr. med. Bernadett Erdmann<br>Klinikum Wolfsburg - Zentrale Notaufnahme<br>Sauerbruchstr. 7, 38440 Wolfsburg<br>bernadett.erdmann@klinikum.wolfsburg.de<br>05361 80-1570<br><br>Prof. Klaus Hahnenkamp<br>Uniklinikum Greifswald - Zentrale Notaufnahme<br>Ferdinand-Sauerbruch-Straße, 17475 Greifswald<br>klaus.hahnenkamp@med.uni-greifswald.de<br>03834 86-5801

	Prof. Harald Dormann<br>Klinikum Fürth - Zentrale Notaufnahme<br>Jakob-Henle-Straße 1, 90766 Fürth<br>zna@klinikum-fuerth.de<br>0911/7580 2810<br><br>Klaus Rupp<br>Techniker Krankenkasse<br>Bramfelder Str. 140<br>22305 Hamburg<br>Klaus.rupp@tk.de<br>040 69091932
31. Angaben zur Eignung der Prüfstelle, insbesondere zur Angemessenheit der dort vorhandenen Mittel und Einrichtungen sowie des zur Durchführung der klinischen Prüfung zur Verfügung stehenden Personals und zu Erfahrungen in der Durchführung ähnlicher Studien	Im Rahmen des Forschungsprogramms „Biomarkers in Cardiology" (BIC) und „Emergency Processes in Clinical Structures" (EPICS) werden im Arbeitsbereich Notfallmedizin seit über 10 Jahren Investigator-initiierte akutmedizinische Forschungsvorhaben in dem herausfordernden Setting einer Notaufnahme durchgeführt. Diese Projekte umfassen multizentrische, auch internationale Forschungsvorhaben, die vom Arbeitsbereich Notfallmedizin federführend entwickelt und durchgeführt wurden. Außerdem werden wichtige Auftragsstudien für akutmedizinische Krankheitsbilder wie das akute Koronarsyndrom, Vorhofflimmern, akute Herzinsuffizienz, Sepsis und exazerbierte COPD durchgeführt.
32. Vereinbarung über den Zugang des Prüfers/Hauptprüfers/Leiters der klinischen Prüfung, zu den Daten und den Grundsätzen über die Publikation.<br>- Publikationen in einer Form, die keinen Rückschluss auf die Person zulässt.	Die Publikation der Studienergebnisse in Fachjournalen erfolgt in einer aggregierten Form, die keine Rückschlüsse auf Einzelpersonen zulässt.
33. Angaben zur Finanzierung der Studie: Finanzierungsquelle (Name und Sitz) und Höhe der Förderung in €.<br>-ggf. Angabe der Kostenstelle zur ILV Abrechnung der Gebühr	Die Studie wird mit 8.273.510,00 € aus öffentlichen Fördermitteln im Rahmen des Innovationsfonds des Gemeinsamen Bundesausschusses finanziert.<br>Kontaktperson:<br>Katja von Storch<br>Gutenbergstraße 13<br>10587 Berlin<br>Telefon: +49 228 3821-2214<br>E-Mail: katja.storch@dlr.de

Name und Unterschrift des/der Antragsteller/s:

Ich versichere hiermit, dass die in diesem Antrag gegebenen Informationen richtig sind. Ich bin der Auffassung, dass es möglich ist, die o.g. Studie in Übereinstimmung mit dem Protokoll und den nationalen Rechtsvorschriften durchzuführen.

Name: Möckel

Vorname: Martin

Adresse: Charité – Universitätsmedizin Berlin

Campus Virchow-Klinikum
Augustenburger Platz 1
13353 Berlin

Position: Ärztlicher Leiter der Rettungsstellen CVK und CCM

Datum: 29.03.2021

Unterschrift:

Univ.-Prof. Dr. med. Martin Möckel
