# Supplementary material for: The Abdominal Pain Unit (APU). Study protocol of a standardized and structured care pathway for patients with atraumatic abdominal pain in the emergency department: A stepped wedged cluster randomized controlled trial
Source: PLoS One. 2022 Aug 24;17(8):e0273115. doi: 10.1371/journal.pone.0273115 (PMC9401147; doi:10.1371/journal.pone.0273115)
Supplement: S2 Protocol — (DOCX) [file pone.0273115.s003.docx]

Application for advice by the ethics committee on the implementation of a medical-scientific project that does not involve the clinical testing of a medicinal product or medical device

| 1. Title of the study | The **A**bdominal **P**ain **U**nit (APU). Study protocol of a standardized and structured care pathway for patients with atraumatic abdominal pain in the emergency department: A stepped wedged cluster randomized controlled trial. |
| --- | --- |
| 2. Ethics Committee application number | **EA2/219/20** |
| 3. Decisions of other ethics committees on the same matter | n.a. |
| 4. Subject of the study and its objectives; statement of the hypotheses, separated into main and secondary hypotheses, and of the clinical parameters (primary and secondary endpoints) against which the hypotheses are to be tested. | Patients with atraumatic abdominal pain have a very broad range of diagnoses, therefore, those patients with atraumatic abdominal pain should receive faster, safer and more targeted care.  To assure high quality of care and potentially reduce mortality, it seems imperative to implement a novel management pathway, which standardizes the process from start of care to final diagnosis, disposition and specific therapy of atraumatic abdominal pain patients in the emergency department (ED). Therefore, a team of multidisciplinary experts has developed the „Abdominal Pain Unit“(APU) treatment process based on the Delphi method. With APU care is structured according to symptoms and processes rather than diagnosis. The implementation of a new structured, symptom-based process is necessary because patients present themselves in the ED primarily with a symptom and not with an already existing diagnosis. In this way, the indication quality of the diagnostics carried out is improved and patients can be treated more quickly and efficiently.  This study aims to improve German ED care for patients suffering from atraumatic abdominal pain. An application-supported pathway for the ED will be implemented, which supports quick, evidence-based, and standardized diagnosis and treatment plans for patients with atraumatic abdominal pain in the ED.  We hypothesize that the App-based APU-process will lead to:  1) Shorter duration of treatment at the ED while improving patient-reported outcomes (assessed as acute pain score or/and patient satisfaction) at discharge from the ED; or an  2) Improvement of patient-reported outcomes (assessed as acute pain score or/and patient satisfaction) at discharge from the ED while measuring a constant duration of treatment; or a  3) Shorter duration of treatment and unchanged patient-reported outcomes (assessed as acute pain score and patient satisfaction) at discharge from the ED.  All three scenarios will be interpreted as improvement of patients care by the new pathway.  Primary outcomes:  Acute pain score at discharge from the ED, patient satisfaction at discharge from the ED, duration of treatment in the ED  Secondary outcomes:  Patient safety, process quality, quality of care |
| 5. Explanation of the significance of the study | The results of this study are of great relevance for the care of patients with atraumatic abdominal pain in the ED.  Atraumatic abdominal pain has a high prevalence (between 2015 and 2017, approx. 18.5% of all patients presenting to Charité’s ED (CCM+CVK) showed the leading symptom of abdominal pain) and a high mortality (intrahospital mortality approx. 5%). Patients with atraumatic abdominal pain can have a very broad range of diagnoses, ranging from flatulencies to more serious underlying diseases, such as an acute pancreatitis.  In view of the demographic situation (increase in older population groups, increase in groups in need of care, multimorbid and partly demented groups), it is to be expected that the number of patients with non-specific abdominal pain symptoms will continue to increase.  Simultaneously, for various reasons, especially economic reasons, a time limit exists on the treatment time per patient in the ED, and additionally, ED staff can be significantly overloaded with work due to the high numbers of patients. These aspects can lead to the danger that serious diagnoses are overlooked (underuse) or that an overdiagnosis and the associated disadvantages (e.g. radiation exposure) can occur. On the patient’s side, dissatisfaction with the treatment can occur  Therefore, there is a need for an evidence-based, standardised care process for patients with atraumatic abdominal pain.  Care processes for patients with acute chest pain, as well as patients with a suspected stroke have already been successfully established within the framework of "Chest Pain Units" and "Stroke Units".  To the best of our knowledge, no validated randomized studies for a care process as proposed in this study (i.e. for the app-supported care process of the "Abdominal Pain Unit") is still lacking. The proposed study aims to close this research gap. |
| 6. Which of the following provisions apply?   1. German medical device act according to § 23b MPG –exception of the clinical trial / examination 2. German radiation protection act & radiation protection ordinance 3. German genetic diagnostics act 4. German data protection law:   - Concrete specification of the responsible body to comply with data protection law (for Charité = -EU- general data protection law (DSGVO), Berlin federal data protection law - BlnDSG).  - If applicable, according to the group of participants additional data protection laws or Data protection laws or BDSG. | Explanation: After examination by multiple authorities, which are listed below, the app is not defined as a medical device, nor does the Medical Devices Act apply.  1.) Clinical Trial Office, BIH, Dr. Uwe Behrens,  2.) Lead of the Ethics Committee of Lageso Berlin, Antje Kettner-Ottilie  3.) Unit for medical devices, Lageso, Inspector medical devices Dr. Matthias Merx,  d) The Berlin federal data protection law applies. The Charité data protection officer will check whether other data protection laws (state data protection laws) might apply. |
| 7. If applicable: designation and characterisation of the test products | No physical product is tested. The APU treatment process is a structured, standardised pathway for the treatment of atraumatic abdominal pain. The individual sections of the treatment process and their representation in the supporting App will be explained below as examples. We then specifically address the role of the APP in the process. For a better understanding, in appendix 4, the extended event-driven process chain (eEPK) of the APU treatment process is illustrated, which was developed by experts in a Delphi process as the basis of our project.  The APU process:  At the beginning of the treatment process, there is the Standard Operation Procedure (SOP, checklist) "Clinical Assessment Shock/Sepsis" (field 3 in the eEPK, appendix 7), which is intended to support the medical staff in recognising vital endangered patients at an early stage.  The SOP includes the following questions for the attending physician: Have you checked blood pressure, heart rate, oxygen saturation, alertness? Is the patient circulatory stable:  Yes? / No?  Assistance is also given for calculating alertness (Glascow Coma Scale) or for calculating clinical scores (in this case qSOFA score for assessing whether sepsis/shock is present).  Once shock has been ruled out according to the appropriate SOP, the basic measures are carried out. These include the patient history and physical examination (field 10), basic laboratory diagnostics (field 14), and the implementation of adequate pain management (field 18). The SOPs stored for this purpose are intended to provide the attending physician with assistance for a possible, efficient procedure.  All SOPs mentioned are part of the treatment process and are displayed informatively via the App.  The basic measures are followed by the SOP "Clinical assessment" (field 23) of the patient, which are presented as an example in the appendix.  In this SOP, it is assessed whether a confirmed or unclear diagnosis is available. Then, on the basis of information collected in the anamnesis, in the physical examination, as well as the laboratory findings that are extracted the local hospital information system (which are not transferred to the app), the physician has to decide, whether a "confirmed diagnosis" or still an "unclear diagnosis" or even a "shock" exists at the time of the renewed clinical assessment.  An example of an SOP is shown in appendix 7.  If there is a confirmed diagnosis, the treatment process leads to the end of treatment, and thus to the SOP "Specific management" (field 23).  If there is no confirmed diagnosis, sonography is suggested to the physician as a further diagnostic test and the following possible further steps of the treatment process (observation, radiological imaging, consultation). We will not go into detail about these further steps here. However, they do not differ in structure and principle from the previously listed SOPs.  At the end of the process, as mentioned above, there is the SOP "Specific Management", which can be used to check whether all information important for the end of treatment has been obtained and the prerequisites for discharge/transfer/intervention/surgery are in place.  Role of the app within the APU treatment process:  The treatment process, in more detail the SOP "Clinical Assessment" (see appendix 7) within the App supports the medical tasks as follows:  - Display of Standard Operating Procedures  - Documentation of time stamps  - Documentation of set "ticks" in checklists  - Calculation of clinical scores (similar and common with/in the hospital information system)  The SOPs mentioned above *explicitly do not* contain any binding requests for action for the physician. In the SOP "Anamnesis", for example, typical symptoms associated with abdominal pain are presented in a tabular form. In the SOP "Laboratory abdominal pain", the physician can read through which laboratory parameters should not be forgotten. However, the physician does not have to select any parameters. Thus, the pathway supported by the APU-App does not compete with the usual procedural instructions of the hospital.  There are no "mandatory fields" in the individual SOPs. The user is always free to decide which aspects of the APU treatment process are to be followed or, if necessary, skipped. The documentation function of the App can be used to record process compliance in this context.  The APU treatment process, which will be evaluated in this study, serves the purpose of "detecting, preventing, monitoring, treating or alleviating disease".  However, the supporting App itself has no influence on the treatment process as such. It merely displays its content and allows documentation of whether the user has taken note of the relevant information. It explicitly does not serve the purpose of device-based detection, prevention, monitoring, treatment or alleviation of diseases. It does not use artificial intelligence and does not make or suggest decisions for the user at any time. The decision and interpretation of findings remains the sole responsibility of the treating medical staff. However, it ensures that the necessary information and findings are available according to the "state of the art".  In summary, the APU process is a treatment procedure that is software-supported via an application ("app") on stationary (e.g. desktop PCs) and/or mobile devices (e.g. tablets).  In APU, treatment is carried out according to the current treatment guidelines and the APU process merely provides a standardised structure of the treatment pathway. The aim is not to overlook any diagnostic and therapeutic steps, similar to a checklist. Equally, however, the aim is not to carry out any unnecessary therapeutic steps or diagnostics. No new diagnostic or therapeutic measures are implemented. Only the treatment pathway itself, which provides a standardised structure for the treatment of atraumatic abdominal pain, is implemented. The treatment thus becomes more structured and safer for the medical staff. The app used in this study, serves as an instrument on which the APU treatment process is displayed |
| 8. Significant results of pre-clinical tests or reasons for not carrying out such tests | Not applicable |
| 9. Essential content and results of previous studies/applications of the products to be tested in the study | We are not aware of any study that establishes and validates an evidence-based, standardised care process for patients with atraumatic abdominal pain.  Although, for example, a clinical algorithm for the diagnosis and treatment of acute abdominal pain in the ED was developed by Trentzsch and colleagues in 2011. However, this clincal algorithm was never tested prospectively, in a RCT.  (appendix 1 (Trentzsch, Werner et al. 2011)).  References:  Trentzsch, H., et al. (2011). "Der akute Abdominalschmerz in der Notfallambulanz – ein klinischer Algorithmus für den erwachsenen Patienten." Zentralbl Chir 136(02): 118-128 |
| 10. Description of planned measures/examination methods and any deviations from the measures/examinations commonly used in medical practice (what is "routine", what is done differently in the study?).  If validated questionnaires are used in the study, please state the name of the questionnaires and where they are published (references).  Please include non-validated questionnaires as an attachment | The present study is a mixed-methods multicenter trial in 10 hospitals with EDs.  Intervention:  Prior to the study, a pilot study will be conducted over a period of 3 months to test study material and processes. Then, all 10 study sites in this multicenter stepped-wedge cluster randomized controlled trial (cRCT), will start in the control phase and will then consecutively (every 4 months 1 cluster (2 study sites) will start the intervention phase) be enrolled into the intervention arm over a trial period of 24 months. 5 cluster with 5 different  Control:  During the control phase, case as usual in the ED is provided to all patients with atraumatic abdominal pain. Current care as usual of atraumatic abdominal pain patients in the ED is not always evidence-based and might differ significantly from hospital to hospital. Furthermore, the accuracy of the implementation of these recommendations for action is often dependent on the experience and intuition of the attending physician.  Deviation from the standard procedure during the intervention phase:  In the APU treatment process, common treatment guidelines are applied without changing their core content.  Patients with atraumatic abdominal pain should receive faster, safer and more targeted care. This is achieved by implementing a new care process that is structured according to symptoms and processes rather than diagnosis.  The app-supported APU-process (in the following and for clarity, the term APU-process will be used) starts with a patient who suffers from atraumatic abdominal pain presenting in the ED. By means of the APU-app, physicians will be supported in making a structured decision for the subsequent diagnostic and treatment process. Eventually, the APU treatment process ends with either the discharge of the patient with a sufficiently accurate diagnosis from the ED or the patient being admitted to another hospital unit for further treatment. Patients with a shock syndrome or sepsis leave the path for special intensive care early.  The first step in the APU treatment process includes a medical history, a medical examination, measurement of blood parameters, and pain management for patients with atraumatic abdominal pain.  In a second step, a re-evaluation will lead to a decision whether the patient will be discharged from the ED to ambulant care (i.e. in case of unsuspicious clinical findings) or if further diagnostic measures have to be taken. Thus, in a third step, the patient will receive a sonography, however, in the case of persistently unclear clinical findings, in a fourth step, either additional imaging methods will be used, such as computer tomography or magnetic resonance imaging, or a multi-disciplinary consultation and if necessary a patient observation for a few hours will be performed. Patients with worsening medical condition will leave the path for intensive care.  The implementation of a new structured, symptom-based process is necessary because patients present themselves in the ED primarily with a symptom and not with an already existing diagnosis. In this way, the indication quality of the diagnostics carried out is improved and patients can be treated more quickly and efficiently.  This treatment process is to be presented with software support using an app. The app serves as a contemporary means of presenting the process and at the same time as documentation software in the context of the application of the new treatment pathway. The app itself has no influence on the care process, nor does it dictate to the physician using the app how the treatment/therapy is to be carried out.  It merely displays its content and documents whether the user has taken note of the relevant information. It expressly does not serve the purpose of device-based detection, prevention, monitoring, treatment or alleviation of diseases. It does not represent artificial intelligence and does not make decisions for the user at any time. The decision is still the responsibility of the attending medical staff. Thus, the app is a documentation software in the context of process optimisation through the APU treatment process.  The focus of this study will be the new APU treatment process and NOT the approval of the app as a medical device, but the evaluation and possibly approval of the new APU treatment process.  The app receives information manually entered by the treating medical staff. This information includes a patient identifier, patient’s name and age.  The aim of the new process is to avoid unnecessary therapeutic or diagnostic steps and, through the standardised structure. Moreover, the novel APU treatment process aims to prevent important findings from being overlooked or urgently needed measures from being omitted. The focus here is on pointing out the various diagnostic and therapeutic measures without making a final recommendation. The APU treatment process aims to be a work through in the treatment process of patients with atraumatic abdominal pain, similar to a checklist.  The process has so far been presented as an event process chain (EPC). The schematic representation can be found in appendix 4  The treatment procedure is thus more standardised and objectified compared to the "routine procedure".  The evaluation of the study follows a mixed methods approach. Quantitative and qualitative primary data will be triangulated with secondary data from the hospital information system and billing data from the participating health insurance funds. Furthermore, expert interviews are planned.  The evaluation is conducted according to five different modules at different points in time:  - Module 1 (patient-reported primary data): Control and intervention groups will be interviewed via tablet computer (if necessary as paper-pencil) upon discharge from the ED (survey t0). If patients cannot be interviewed in the ED (e.g. due to a transfer to the surgery room), a follow-up survey can be conducted up to 72 hours after the initial contact in the ED on the treatment ward to which the patient is transferred. After 30 days, a telephone follow-up or an online survey is conducted (t1). Patients will be contacted in advance by phone or e-mail to remind them of the follow-up. The analyses include current pain score (numerical rating scale (NRS)), satisfaction with own health and quality of life (items taken from EUROHIS-QOL-8; appendix 2: (Schmidt, Mühlan et al. 2005)), socio-demographic data, as well as patient satisfaction ((ZUF-8) appendix 3 (Schmidt, Wittmann et al. 2002)) and life satisfaction ((Short Scale Life Satisfaction-1 (L-1) appendix 4 (Beierlein et al. 2014.)). See appendix t0 and t1 questionnaire.  - Module 2 (primary data of care): Clinical parameters (e.g. vital signs, previous illnesses, onset of pain, accompanying symptoms, medication history) and the duration of the stay in the ED are recorded for each patient. The data collection takes place without participation of the patients after their discharge from the ED.  - Module 3 (secondary hospital data): For patients with abdominal pain during the study period, the data available for them is extracted from the hospital information system (HIS). The HIS data include, for example, blood parameters, diagnostic examinations and results, diagnoses, procedures, operations and the hospital history (wards, intensive care unit, length of stay, complications, mortality, DRGs, billing figures) of the patients. The data collection is carried out by study staff without the involvement of the patients after their discharge from the ED.  - Module 4 (secondary data of the health insurance company): By declaring their participation in the study, patients agree to transmission of their health available billing data from the statutory health insurance company to the evaluating institute in accordance with §75 of the German Social Code Book V. The transmitted data includes both, the outpatient and inpatient service areas one year before the index stay as well as a period of 30 days after the initial contact. The data can be analysed longitudinally. Important parameters are the development over time in the form of disease progression, mortality and the services used. Furthermore, all patients treated during the study period are identified in the data of the participating statutory health insurance companies and also analysed internally (at the participating health insurance company) with regard to the costs incurred before and after hospitalisation.  - Module 5 (expert interviews, participant observation): Within the framework of participant observation, 5 patients in each of 5 hospitals will be followed after the establishment of the abdominal pain units over the period of their ED stay (n=25). The observation starts with the start of treatment and ends with the discharge from the ED. The observations, which also include the spoken word, are systematically documented in the form of protocols. Furthermore, 2 expert interviews will be conducted per hospital after the introduction of the Abdominal Pain Unit within the project period (total n= 20). The expert interviews will be conducted as semi-structured interviews, recorded on tape and transcribed.  References:  Schmidt, J., et al. (2002). Fragebogen zur Messung der Patientenzufriedenheit. Diagnostische verfahren in der psychotherapie. Göttingen: Hogrefe.  Schmidt, S., et al. (2005). "The EUROHIS-QOL 8-item index: psychometric results of a cross-cultural field study." European Journal of Public Health 16(4): 420-428.  Beierlein et al. (2014). Eine Single-Item-Skala zur Erfassung der Allgemeinen Lebenszufriedenheit: Die Kurzskala Lebenszufriedenheit-1 (L-1). GESIS-Working Papers 2014 \| 33. GESIS: Köln. |
| 11. Evaluation and consideration of foreseeable risks and disadvantages of study participation with regard to expected benefits for study participants and persons who will become ill in the future (risk-benefit assessment). | |
| a. Medical benefit to be tested for the study participants (individual benefit for the patient as an individual) | It is assumed that by applying the APU treatment process, the quality and preciseness of performed diagnostics can be improved. Moreover, patients can be treated faster and more efficiently.  The individual benefit for the patient would be a reduction of the time spent in the ED, a faster and more adequate pain management, as well as an increased satisfaction with the treatment.  Module 5: The individual benefit for study participants (experts) in expert interviews would be a possible reflection and improvement of the application and work processes in connection with the APU app. With regard to the participating patient observations, no direct individual benefit is to be expected. |
| b. Medical benefit to be tested for persons with the disease in the future (benefit to the community) | For future patients, the benefits would also be reflected in faster and more efficient care for patients with atraumatic abdominal pain. It can be assumed that the aforementioned positive effects will be further strengthened with the increasing establishment of the APU treatment process and thus waiting times and pain levels will decrease and patient satisfaction will increase.  In addition, the results of this study create a scientific basis for further optimisation of the care of abdominal pain patients in the ED. |
| c. Risks and burdens for study participants (tob e listed seperately) | The application of the APU treatment process itself does not pose any additional risk, as the APU treatment process merely brings common treatment guidelines into a standardised structure without changing their core or bringing in new procedures that have not yet been established clinically.  These treatment guidelines include, for example:  - Clinical examination  - Medical history  - Laboratory tests  - Imaging procedures (e.g. sonography, CT)  - Specialist consults  - Pain management  - Re-evaluation and therapy of shock, sepsis at specified times  Thus, only procedures that are already established in clinical routine are applied.  There is a potential risk that the APU treatment process could lead to technical problems in the implementation of the process or to confusion among the staff using the app, thus delaying treatment decisions. In order to safeguard that the app as user-friendly as possible and in order to avoid risks (e.g. technical problems), the app will be extensively tested in a clinical setting in a pilot phase and the application will be optimised.  Otherwise, there is no immediate risk for participants by participation in the study and no harm is to be expected. |
| 12. Measures to be taken to manage the risk | In order to keep the above-mentioned risks as low as possible, it is planned to test the APU application ("app") extensively before the first use on the patient, to ensure the interoperability of the app with different devices and to train the participating staff extensively in the use of the APU treatmen process including the work with the app. The app will continue to be tested and further optimised in a pilot phase in a clinic. |
| 13. Termination criteria | If participation in the study represents an unreasonable burden for patients or experts, participation should be discontinued. Participants are informed that they can withdraw their consent and discontinue the study at any time without giving reasons. |
| 14. Number, age and gender of the persons concerned | Study population:  All adult patients of all genders who present in the specified period with the inclusion and exclusion criteria specified in point 16, estimated at 2.000 patients.  Module 5: For semi-structured interviews, n=20 adult experts of all genders will be interviewed.  For the participant observations, n=25 adult patients of all genders will be observed. |
| 15. Biometric planning with indication of statistical methodology, including power calculation.  Indication of the statistician(s)  (if advice is given by the Institute of Biometry of the Charité, a signature must be inserted) | Justification of the selected case numbers/sample sizes (power calculation).  In the evaluation of a new form of care with a broad focus on improving patient care, there is no single research hypothesis to be tested, but rather a series of combined endpoints (process times, patient-related endpoints). Therefore, no classic sample calculation is possible, as it is usual for confirmatory effectiveness studies. Furthermore, the achievable number of patients is limited by the study centers and the expected proportion of emergency patients with atraumatic abdominal pain. The justification of the sample size is therefore based on feasibility considerations and accordingly a power analysis was performed for relevant endpoints of evaluation modules 1 and 2. A total achievable sample size of 2.000 patients is expected. With a conservative estimate, we expect a loss to follow-up of 15%, since the time of transfer for patients, as well / or the time of outpatient discharge is not always predictable. Moreover, the recording of the endpoints may not be possible in the case of emergency transfers. This means that the expected number of evaluable patients is reduced to at least n=1.700. For the correction of the stepped-wedge design according to Hemming (appendix 4: (Hemming and Taljaard 2016) the following assumptions were made: there are random cluster effects, fixed time effects, but no interactions between cluster and time. Intracluster correlation coefficients (ICC) are usually between 0.01 and 0.02 in human studies (appendix 5: (Killip, Mahfoud et al. 2004). Choosing the conservative variant of an ICC of 0.02, which leads to the highest SWD correction factor on this interval, we obtain a correction factor of 2.71. Applying this correction factor results in an effective total number of cases corresponding to an RCT design of n=627, i.e. n=313 per treatment arm. For the power estimates, the significance level for 3 parallel tests according to Bonferroni is adjusted to α*=0.05/3=0.0167. For the above-mentioned primary endpoints under the respective assumed effect sizes δ and α*, the following numbers of cases would result for an RCT: 1) for δ=0.341 in a 2-sided t-test, n=183 per group, 2) for δ=0.0863 in a Chi² test, n=105 per group, and 3) for δ=0.499 in a 2-sided t-test, n=86 per group are required. The power calculations lead to the result that the study is sufficiently powered for all 3 endpoints with an RCT-equivalent of n=313 patients per treatment arm when testing the 3 test hypotheses defined above. Specifically, a power of 1) 96%, 2) 99% and 3) 99% could be achieved for the assumed effect sizes with the achievable number of cases n=313 per treatment arm corrected for SWD. Especially for the "patient reported outcomes" of endpoints 2 and 3, the assumed number of cases and thus the achievable power would make the adjustment of the evaluations for do not exclude potential confounders such as age and gender from the outset. Power calculations and power estimates were performed with nQuery Advisor 7.0.  Statistician:  Dr. Dörte Huscher, Institut für Biometrie und Klinische Epidemiologie, Charité – Universitätsmedizin Berlin  References:  Hemming, K. and M. Taljaard (2016). "Sample size calculations for stepped wedge and cluster randomised trials: a unified approach." Journal of Clinical Epidemiology 69: 137-146.  Killip, S., et al. (2004). "What is an intracluster correlation coefficient? Crucial concepts for primary care researchers." Ann Fam Med 2(3): 204-208. |
| 16.  a. Presentation and, if applicable, explanation of the inclusion and exclusion criteria | Inclusion criteria:  Adult patients with atraumatic abdominal pain  Capacity to consent (self or through a legal representative / carer / guardian)  Module 5: For semi-structured interviews, staff members (e.g. physicians) of the respective ED who have used the novel APU app will be interviewed.  For the participant observations, individual patients will be observed.  Exclusion criteria:  Obvious trauma or accident as cause of abdominal pain  Presence of shock or sepsis (qSOFA ≥ 2) on admission to the emergency department.  Module 5: semi-structured interviews: Experts who have not used the APU app.  Participatory observations: None. |
| b. **Study information** (information about who informs - verbally and written - and indication of how much time remains between information and consent (=reflection period) (written information as an attachment). | Due to the heterogeneity of the treatment processes of patients with atraumatic abdominal pain in the ED, the reflection period cannot be quantified. For this reason, potential study participants are first informed about the study verbally and in written form by the study team after the initial assessment in triage. The reflection period continues until contact is made with the attending physician. The treating physician informs the patient again and gives the patient further time to think about participation until the start of treatment. Study participants have sufficient time to ask questions. A longer reflection period cannot be granted, as the intervention is linked to the treatment in the ED and the study is not directly invasive in nature. Patients are informed about the risks (see point 11c).  Module 5: Experts will be informed in written form with study information about potential participation in semi-structured interviews (e.g. via e-mail or in study kick-off meetings) (see appendix X). A reflection period between information and consent will at least be 24 hours. |
| c. **Informed consent** (written as appendix) | A consent form is used for the study. In it, the participants declare their voluntariness and their consent to participate in the study (see Appendix A).  A separate consent form will be used for the expert interviews in Module 5 (see Appendix X). |
| d. If applicable, information and consent of the legal representative (if applicable, also description of the procedure for establishing judicial care). | For patients with a legal representative, this representative will be informed and a separate section in the consent form will be used for the consent. The legal representatives of the participants declare their voluntary participation and consent to participate in the study (see appendix A). |
| 17. Recruitment strategies | The identification of study participants takes place in the ED within the framework of initial nursing care. In the period from the start of the project to the start of the intervention, control group patients are recruited, who receive care as usual. With the implementation of the new form of care, patients are recruited for the intervention group. Identification is based on the leading symptom on presentation in the ED (i.e. abdominal pain). No further measures are planned.  Module 5: The recruitment of experts for the semi-structured interviews will take place during staff trainings / training of users of the APU app or by e-mail as an invitation letter to participate in the study with study information (e.g., information on the objective, background, and the results of the study): Information on the objective, background, data protection regulations of the study, as well as an explanation of the interview procedure).  For participant observations of the treatment process of patients, see the identification of patients in the ED. |
| 18. If applicable: Reason for inclusion and demonstration of therapeutic benefit for persons who are minors and/or unable to consent. | As far as possible, all patients with atraumatic abdominal pain should be able to participate in the study in order to be able to examine a representative sample that can be extrapolated into subgroups in analyses. For patients with a legal representative, this representative will be informed and a separate section in the consent form will be used for the consent.  In this case, a copy of the power of attorney of the legal representative has to be obtained beforehand. |
| 19. Relationship between study participant and study physicians (Is the study physician also the treating physician?) | Study physicians can also be the treating physicians of the study participants. Admission to the study takes place after identification by the study physician as part of the initial nursing care in the ED. |
| 20. Declaration on the involvement of persons possibly dependent on the sponsor | No sponsor-dependent individuals will be included in the study. |
| 21. Measures, which determine whether a study participant can participate in another study at the same time / or whether it is allowed to include participants in the present study if participation happened in a previous study.  Is participation in multiple studies possible? | Participation in another study is possible, as long as investigated endpoints of the present study are not confounded by participation in another study. |
| 22. If applicable: remuneration or reimbursement of study participants (amount, what should be paid for?) | Patients do not receive a fee/reimbursement for participation in this study. No costs will be made by patients for their participation. |
| 23. If applicable: Plan for the further treatment and medical care of the participants after the end of the study. | Participating and non-participating persons are treated according to valid guidelines. Depending on the degree of severity, patients are either treated on an outpatient basis or admitted to hospital. Medical care/treatment is also ensured after the end of the study within the structures of the participating clinics. |
| 24. If applicable: Insurance of study participants (Confirmation of insurance and conditions of insurance, insurer, scope of insurance, term of insurance) | In our view, additional insurance for the study participants is not necessary. The liability insurance of the participating clinics is considered sufficient. |
| 25. Documentation procedure:  - If applicable: reference to CRFs  - Indication of data to be collected  - Sample handling  - Retention / archiving (incl. time limits)  - Access to data and samples | The data collection and the selection of the instruments to be used are based on international standards and are developed in a multidisciplinary process. The final data collection forms will be submitted later.  Collection of primary data happens decentralised in 10 clinics for 2.000 patients. The primary data are to be recorded in electronic case report forms (CRFs) and subsequently merged centrally. The consolidation is done via the data entry and management software secuTrail. A scientific documentalist is planned for the development of data collection instruments, electronic implementation, pseudonymisation, data merging and extraction, data monitoring, data cleaning and plausibility checks. The described evaluation-specific data on emergency admissions and hospital stays will be collected (primary data), and secondary data will be processed. Secondary data will be made available both, the hospital information systems as well as the participating health insurance company. Within the framework of the project, a central, relational database will be set up in which primary and secondary data will be made available in pseudonymised form. The scientific staff of the participating evaluating institutes, as well as the biometrician of the project, are involved in all phases of data collection, processing and evaluation. Quality assurance measures include the training of staff in data collection and technical support for the implementation of electronic data collection on site in the participating EDs, as well as monitoring of data entry and quality on site and centrally.  Two study physicians per study center (n=20) who have used the APU app / process will be interviewed with semi-structured interviews. The only documents recorded are the consent forms for interview participation. Study physicians will be recruited during staff training for the APU app or by e-mail as a letter inviting them to take part in the study with study information (e.g. information on the objective, background and the study protocol): Information about the objective, the background, data protection regulations of the study, as well as an explanation of the interview procedure). The interview will be recorded as an audio file. No personal identifying data of the physicans (or of patients through interview answers) will be collected. The audio files will be transmitted to the transcription company in accordance with data protection regulations, transcribed and transmitted back to the Institute of Medical Sociology and Rehabilitation Research. Interview transcripts are stored in anonymized form with restricted access on the servers of the Charité. The audio files of the interviews will be deleted after receipt of the interview transcripts by the Institute of Medical Sociology and Rehabilitation Research or after completion of the transcription process by the contracted transcription company. A commissioned processing agreement is concluded between the transcription company and Charité in accordance with Art. 28 DSGVO to carry out the transcription of the audio files. Access to this data is only permitted to the responsible staff member in the APU project.  For the participatory observations, n=25 patients will be observed during ED treatment. All data will be collected anonymously by study staff from the Institute of Medical Sociology and Rehabilitation Research, i.e. no personal data will be entered into the observation records, but the application of the new form of care in the "real-life" setting, as well as possible influencing contextual factors (e.g. ED procedures, communication structures, patients, staff, etc.) will be observed and recorded. The anonymous protocols are stored and analysed at the Institute for Medical Sociology and Rehabilitation Research.  Only the principal investigator of the study, as well as the staff in charge of data management, have access to the study data.  The study data will be analysed for up to 2 years after the end of the study funding.  The study data will then be kept for 10 years and then deleted. A corresponding data protection concept is being developed with the official data protection officer of the Charité and other responsible bodies. |
| 26. If applicable: description of how the health status of affected healthy persons is to be documented. | Not applicable. |
| 27. If applicable: methods to identify, document, and report adverse events (when, by whom and how ?). | Adverse events will be recorded during the index stay and telephone follow-up. All adverse events will be reported to the principal investigator within 3 working days. Further reports will be made as required |
| 28. Procedure for protecting the confidentiality of the stored data, documents and, if applicable, samples, description of the pseudonymisation or anonymisation of the data and samples of study participants (initials and date of birth as coding scheme are not permitted!).  - Description of the separation of  medical records, study documentation and allocation of personal data  - Designation of access rights including access to participant identification lists during and after the study  - Detailed specification of the procedures for  transmission, encryption, restriction of processing (blocking) and deletion (including details of the network structure used, if any, and servers used).  -If applicable, access to identifying data for legally authorised auditors (third parties) for the purpose of inspection of the files | The study participants are assigned an automatically generated pseudonym.  The participant identification list is kept locked in the rooms of the respective study teams. Only personnel involved in the study and the study leaders on site have access to these documents. After the study has been conducted, the participant identification list will be stored in a lockable steel cabinet at the site of the study leader for the legally required period of 10 years.  Module 5:  Expert interviews are pseudonymised, as no personal data are collected. After deletion of the audio files, the transcripts are anonymous.  Participant observations are collected anonymously.  The study data (expert & patient data) will NOT be passed on to third parties. |
| 29. Declaration of compliance with data protection  - Assurance that all data collected and stored about the participants will be will be treated confidentially (e.g. medical confidentiality)  - Assurance that the identifying data will be disclosed only to the principal investigator or employees appointed / authorized by him/her.  - Indication of the measures taken to ensure  confidentiality  - Measures to ensure data protection  transmission of data which cannot be which do not allow third parties to establish a personal reference.  - Information on information, revocation, correction and deletion options,  - measures to safeguard the rights of the participants.  - If transfers to non-EU countries are intended: Measures for compliance with data protection (e.g. existence of an adequacy decision by the EU Commission or explicit consent of the participants to such transfers). | All data collected and stored about participants / experts will be treated confidentially (data secrecy and medical confidentiality).  The identifying data are only accessible to the principal investigator and the employees appointed by him/her.  The right to information exists for all collected study data. This means that the participant has the right to gain information and correction or deletion of his/her personal study data at any time. |
| 30. Names and addresses of institutions involved in the study as study center or study laboratory, as well as the principal investigator and the study physicians  - Information on external service providers involved with details of the data access option | Study center:  Charité Universitätsmedizin Berlin  Arbeitsbereich Notfall- und Akutmedizin CVK, CCM  Augustenburger Platz 1  13353 Berlin  Principal investigator/head of the trial and responsible physician:  Prof. Dr. Martin Möckel  030 450 553203  martin.moeckel@charite.de  Deputy head of the trial and epidemiologist in charge:  Prof. Dr. Anna Slagman  030 450 565659  anna.slagman@charite.de  Physicians:  PD Dr. Undine Gerlach-Runge  030 450 531000  undine.gerlach@charite.de  Britta Stier  030 450 631317  britta.stier@charite.de  Dr. Lukas Helbig  030 450 631356  lukas.helbig@charite.de  Myrto Bolanaki  030 450 665653  myrto.bolanaki@charite.de  Dorothee Riedlinger  030 450 631316  dorothee.riedlinger@charite.de  Rebecca Resendiz Cantu  030 450 631338  rebecca.resendiz@charite.de  Other study centres and directors of studies:  Cooperation:  Dr. med. Maik Kilian  Evangelische Elisabeth Klinik Berlin  Lützowstraße 26, 10785 Berlin  info.elisabeth@pgdiakonie.de  030 2506-1  Prof. Christian Wrede  Helios Klinikum Berlin Buch Notfallzentrum  Schwanebecker Chaussee 50, 13125Berlin  christian.wrede@helios-gesundheit.de  030 94 01-54700  Dr. med. Markus Wehler  Uniklinikum Augsburg - Zentrale Notaufnahme  Stenglinstr. 2, 86156 Augsburg  sekretariat.noa@uk-augsburg.de  0821 400-3876  Prof. Christoph Dodt  München Klinik Bogenhausen  Englschalkinger Straße 77, 81925 München  [christoph.dodt@klinikum-muenchen.de](mailto:christoph.dodt@klinikum-muenchen.de)  089 9270-3269  Prof. Wilhelm Behringer  Uniklinikum Jena - Zentrale Notaufnahme  Am Klinikum 1, 07747 Jena  [wilhelm.behringer@med.uni-jena.de](javascript:sendEmail('wilhelm.behringer',%20'med',%20'uni-jena.de',%20''))  03641 9-322001  Dr. med. Frank Wösten  Klinikum Bremen Nord, Interdisziplinäre Notaufnahme  Hammersbecker Straße 228, 28755 Bremen  [frank.woesten@gesundheitnord.de](mailto:Frank.Woesten@gesundheitnord.de)  0421 6606-1950  Dr. med. Bernadett Erdmann  Klinikum Wolfsburg - Zentrale Notaufnahme  Sauerbruchstr. 7, 38440 Wolfsburg  bernadett.erdmann@klinikum.wolfsburg.de  05361 80-1570  Prof. Klaus Hahnenkamp  Uniklinikum Greifswald - Zentrale Notaufnahme  Ferdinand-Sauerbruch-Straße, 17475 Greifswald  [klaus.hahnenkamp@med.uni-greifswald.de](javascript:linkTo_UnCryptMailto('ocknvq,mncwu0jcjpgpmcorBogf0wpk/itgkhuycnf0fg');)  03834 86-5801  Prof. Harald Dormann  Klinikum Fürth - Zentrale Notaufnahme  Jakob-Henle-Straße 1, 90766 Fürth  [zna@klinikum-fuerth.de](mailto:zna@klinikum-fuerth.de)  0911/7580 2810  Klaus Rupp  Techniker Krankenkasse  Bramfelder Str. 140  22305 Hamburg  [Klaus.rupp@tk.de](mailto:Klaus.rupp@tk.de)  040 69091932 |
| 31. Information on the suitability of the trial site, in particular on the adequacy of the resources and facilities available there, as well as on the personnel available to conduct the clinical trial and on experience in the conduct of similar trials. | Within the framework of the research programmes "Biomarkers in Cardiology" (BIC) and "Emergency Processes in Clinical Structures" (EPICS), investigator-initiated acute medical research projects have been carried out in the challenging setting of an ED for over 10 years. These projects include multi-center trials, as well as international research projects, which were developed and carried out under the leadership of the Department of Emergency Medicine. In addition, important commissioned studies are conducted for acute medical conditions such as acute coronary syndrome, atrial fibrillation, acute heart failure, sepsis and exacerbated COPD. |
| 32. Agreement on access by the investigator/principal investigator/lead investigator to the data and the principles on publication.  - Publications in a form that does not  inference about the person | The publication of the study results in specialist journals takes place in an aggregated form that does not allow any conclusions to be drawn about individuals |
| 33. Information on the funding of the study:  Source of funding (name and location) and amount of funding in €.  -If applicable, indication of the cost centre for the ILV accounting of the fee. | The study is financed with € 8,273,510.00 from public funding within the framework of the innovation fund of the Federal Joint Committee.  Contact:  Katja von Storch  Gutenbergstraße 13  10587 Berlin  Telefon: +49 228 3821-2214  E-Mail: katja.storch@dlr.de |

Name and signature by principal investigator:

I hereby certify that the information provided in this application is correct. I believe that it is possible to conduct the above study in accordance with the Protocol and national legislation

Name: Möckel

First name: Martin

Adress: Charité – Universitätsmedizin Berlin

Campus Virchow-Klinikum

Augustenburger Platz 1

13353 Berlin

Position: Medical director of the ED (CVK and CCM)

Date: 29.03.2021

Signature:

Univ.-Prof. Dr. med. Martin Möckel
