# Supplementary material for: The Abdominal Pain Unit (APU). Study protocol of a standardized and structured care pathway for patients with atraumatic abdominal pain in the emergency department: A stepped wedged cluster randomized controlled trial
Source: PLoS One. 2022 Aug 24;17(8):e0273115. doi: 10.1371/journal.pone.0273115 (PMC9401147; doi:10.1371/journal.pone.0273115)
Supplement: Attachment — Submitted filename: Response to Reviewers.docx [file pone.0273115.s005.docx]

**Response to review on manuscript “The Abdominal Pain Unit (APU). Study protocol of a standardized and structured care pathway for patients with atraumatic abdominal pain in the emergency department: A stepped wedged cluster randomized controlled trial.” (PONE-D-21-28548)**

We would like to thank the reviewer for his/her thorough review of our manuscript and the constructive, relevant remarks and suggestions. In the following, we will try to explain how we solved remarks indicated by the reviewer. The remarks of the reviewer are depicted first, followed by our responses in italics.

**Reviewer's comments:**

**Reviewer #2:**

A minor comment,... The authors focussed on pain control, patient satisfaction and time in the ED. However, how will this insure that improving these outcomes also will improve standard quality of care outcomes? Please address this in the paper.

*We thank the reviewer for his/her critical thoughts on the outcomes of the APU study and the improvement of quality of care standards. The APU process is no standardized care process, but the project aims to implement a standardized process to quickly diagnose atraumatic abdominal pain in a symptom-based manner. Due to the very heterogeneous nature of the symptoms of abdominal pain, yet no standardized quality of care outcomes/ key indicators exist. For this reason, we aim to mirror an improvement of care with the three primary outcomes (i.e. acute pain, duration of treatment in the ED, and patient satisfaction). Moreover, we also assess quality of care / patient safety and process quality outcomes (e.g. mortality, course of treatment, ICU stay, process times) in the APU study, as mentioned in the manuscript in Table 1 ‘secondary outcomes’ (see yellow highlighted text, page 8 in revised manuscript).*

*We hope that we adequately addressed all concerns and we would like to thank the editor and the reviewer once again for the helpful comments and suggestions. We would, however, be happy to consider further specific suggestions you might have.*
